# Volume-Conducted Origin of the Field Potential at the Lateral Habenula

**DOI:** 10.3389/fnsys.2019.00078

**Published:** 2020-01-08

**Authors:** Nicolas Iván Bertone-Cueto, Julia Makarova, Alejo Mosqueira, Demian García-Violini, Ricardo Sánchez-Peña, Oscar Herreras, Mariano Belluscio, Joaquin Piriz

**Affiliations:** ^1^Grupo de Neurociencia de Sistemas, Instituto de Fisiología y Biofísica “Houssay” (IFIBIO “Houssay”), Universidad de Buenos Aires, CONICET, Buenos Aires, Argentina; ^2^Instituto Cajal, CSIC, Madrid, Spain; ^3^Instituto Tecnológico de Buenos Aires, CONICET, Buenos Aires, Argentina

**Keywords:** lateral habenula, field potential, volume conduction, hippocampus, theta rhythm

## Abstract

Field potentials (FPs) are easily reached signals that provide information about the brain’s processing. However, FP should be interpreted cautiously since their biophysical bases are complex. The lateral habenula (LHb) is a brain structure involved in the encoding of aversive motivational values. Previous work indicates that the activity of the LHb is relevant for hippocampal-dependent learning. Moreover, it has been proposed that the interaction of the LHb with the hippocampal network is evidenced by the synchronization of LHb and hippocampal FPs during theta rhythm. However, the origin of the habenular FP has not been analyzed. Hence, its validity as a measurement of LHb activity has not been proven. In this work, we used electrophysiological recordings in anesthetized rats and feed-forward modeling to investigate biophysical basis of the FP recorded in the LHb. Our results indicate that the FP in the LHb during theta rhythm is a volume-conducted signal from the hippocampus. This result highlight that FPs must be thoroughly analyzed before its biological interpretation and argues against the use of the habenular FP signal as a readout of the activity of the LHb.

## Introduction

The field potential (FP) is a signal generated by the spatiotemporal integration of extracellular currents produced by neuronal activity. Therefore, it is particularly useful to detect synchronized activity during neuronal network oscillations. Since FP rhythms reflect population activity, they could be used as low dimensional signals to infer internal brain states. A widely studied pattern of the FP is the theta rhythm, an FP oscillation in the 4- to 10-Hz frequency range, prominent in the hippocampus during certain tasks such as locomotion or attention (Lisman and Jensen, 2013). It has been proposed that the theta rhythm provides temporal windows in which neurons from the hippocampus and other brain regions coordinate as functional ensembles, allowing the integration of sensory inputs and internal representations during memory formation and retrieval (Hasselmo, 2005; Jensen and Lisman, 2005; O’Keefe and Burgess, 2005). Indeed, such synchronization has been related both to positive and negative motivational learning (Seidenbecher et al., 2003; Narayanan et al., 2007; Lesting et al., 2011; Bazelot et al., 2015; Wikenheiser and Redish, 2015).

The lateral habenula (LHb) is a small brain structure located dorsal to the thalamus that encodes motivational values (Proulx et al., 2014). It receives projections from limbic structures and the basal ganglia and projects to midbrain monoaminergic nuclei, over which it exerts a powerful inhibitory influence. The LHb is generally activated by aversive stimuli, and its activation is sufficient to drive place or stimulus aversion (Shabel et al., 2012, 2014; Stamatakis and Stuber, 2012). Much evidence indicates that the activity of the LHb is required for hippocampus-dependent contextual learning (Tomaiuolo et al., 2014; Mathis et al., 2015; Chan et al., 2017). Thus, it has been shown that the inactivation of the LHb selectively undermines the temporal stability of inhibitory avoidance memory (Tomaiuolo et al., 2014) and impairs learning and retrieval in different spatial tasks (Goutagny et al., 2013; Mathis et al., 2015). Despite the evidence linking the LHb to hippocampus-dependent learning, there is little information about how these two structures communicate. The LHb receives direct projections from the pacemaker regions of the hippocampal theta, such as the diagonal band of Broca (DBB, Aizawa et al., 2013) and the medial septum (MS, Herkenham and Nauta, 1977), suggesting that its activity could be integrated into the hippocampal network by becoming entrained to the theta rhythm. Functional support for this hypothesis comes from studies showing that FP activity measured at the LHb is synchronized with the hippocampal theta rhythm in an object recognition task (Goutagny et al., 2013; Baker et al., 2015). Furthermore, it has been reported that theta power of the FP recorded from the LHb increases when a rat approaches a reward in an eight-arm maze (Baker et al., 2015). However, a conceptual warning should be made about the biophysical basis behind those observations. Structures with no laminar organization, such as the LHb, are expected to generate low power FPs. In contrast, structures with a laminar organization such as the hippocampus are capable to generate high-amplitude FPs. In addition, structures whose local FPs are coherent over large areas are candidates to propagate them over surrounding structures through volume conduction (Lorente de No, 1947; Nunez and Srinivasan, 2009), and this could be even more evident if those structures are unorganized (Torres et al., 2019). Since the LHb is a small unorganized structure that lies just ventral to the dorsal hippocampus, the true origin, i.e., the current generator, of the FP recorded at the LHb deserves a deeper analysis before further interpretations are made.

In this work, we performed electrophysiological recordings in urethane-anesthetized rats and FP modeling to investigate the biophysical nature of the FP recorded at the LHb and its relationship with FP at the hippocampus. Our experimental and numerical evidence indicate that the FP recorded in the LHb is not local; rather, it is volume conducted from the hippocampus. In addition, we found a significant fraction of LHb neurons with their activity locked to hippocampal theta. Remarkably, our model predicts that the theta input to individual LHb cells does not contribute to the extracellular theta. Our results bring attention to a direct interpretation of FP recordings in structures where its origin is not totally understood.

## Results

### Characterization of Theta Rhythm at the LHb and Hippocampus

**FIGURE 1:**
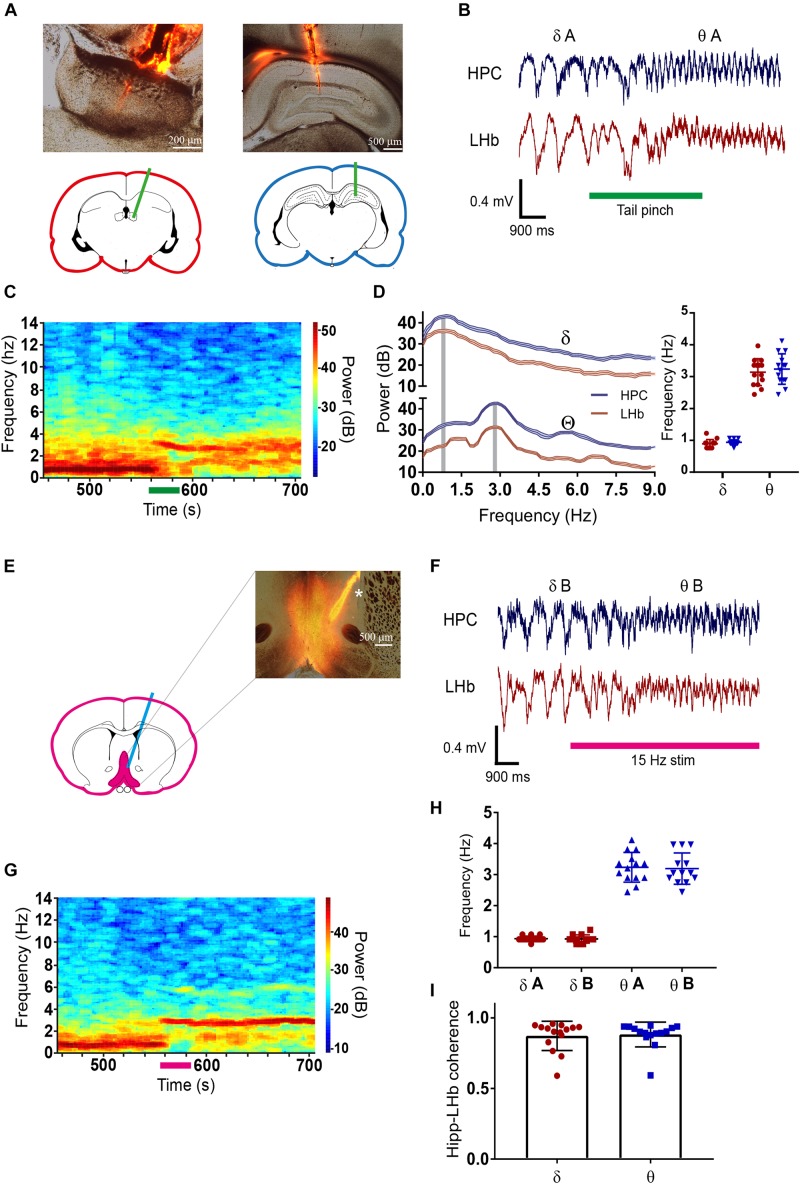
**(A)** Photomicrograph of the electrode location at the lateral habenula (LHb) (left) and the hippocampus (right). Electrodes were labeled with DiI to visualize their position by red fluorescence. Pictures are merged images of the transmitted light and the red fluorescence pictures. **(B)** Example of simultaneous field potential (FP) recording at the LHb and the hippocampus during tail pinch induced transition from delta (δA) to theta (θA). **(C)** Heat plot of the fast Fourier transform (FFT) of the FP at the hippocampus during the tail pinch-induced transition from delta to theta. Before the tail pinch FP was dominated by low frequencies, tail-pinch induced transition to theta range frequencies that persisted afterward. **(D)** Left average FFT of delta and theta periods at the LHb and the hippocampus, gray-shaded columns illustrate maximal frequencies for each state. Right, quantification of maximal frequencies for delta and theta at LHb and the hippocampus (delta state: 0.94 ± 0.08 and 0.89 ± 0.13 for the hippocampus and LHb, respectively; theta state: 3.24 ± 0.48 and 3.14 ± 0.42 for the hippocampus and LHb, respectively, *N* = 14 for all conditions). **(E)** Photomicrograph of the DBB/MS showing the infection with oChIEF-tdTomato encoding AAV and the tract of optic fiber used to light stimulation (^∗^labeled with DiI). Pictures are merged images of the transmitted light and the red fluorescence pictures. **(F)** Example of simultaneous FP recording at the LHb and the hippocampus during the transition from delta (δB) to theta (θB) induced by light stimulation of the DBB/MS complex in an oChIEF-tdTomato-infected animal. **(G)** Heat plot of FP FFT at the hippocampus during the transition from delta (δB) to theta (θB) induced by light stimulation of the DBB/MS complex in an oChIEF-tdTomato-infected animal. Before the tail pinch FP was dominated by low frequencies, 15 Hz light stimulation induced transition to theta range frequencies that persisted after stimulation finished. **(H)** Quantification of delta and theta frequencies at the hippocampus in experiments in oChIEF-tdTomato-infected and non-infected animals. There were no differences in delta and theta frequencies between both groups (mean frequencies for oChIEF: 0.93 ± 0.14, *N* = 13 for delta and 3.19 ± 0.4 Hz, *n* = 13 for theta; the non-infected group is the hippocampus group in **(D)**. **(I)** Coherence between hippocampal and LHb FP’s at delta and theta. For all figures data represent mean ±SD.

To analyze the relationship between neuronal activity at the LHb and at the hippocampus, we performed simultaneous extracellular recordings of the FP in both structures in urethane-anesthetized rats (Figure 1A). As expected, the FP was dominated by a slow rhythm in the delta frequency range (0.5–2 Hz) both at the hippocampus and at the LHb (Figures 1B–D). Upon physical stimulation by a tail pinch, the FP of both structures entered in a higher frequency theta-like rhythm centered at 2.5–4 Hz (Figures 1B–D), coincident with the frequency of the theta rhythm reported by previous studies in urethane anesthetized animals (Kramis et al., 1975). Remarkably, the dominant frequencies of delta and theta were similar both at the hippocampus and at the LHb [Figure 1D, right; repeated measures two-way ANOVA: factor brain state: *F*(1,26) = 368.3, *p* < 0.001; factor brain region: *F*(1,26) = 0.636, N.S.; interaction *F*(1,26) = 0.098, N.S.]. To avoid noxious stimuli, which by themselves could modify neuronal activity at the LHb (Benabid and Jeaugey, 1989; Gao et al., 1996), and to get a better temporal control over delta/theta transitions, we also induced theta rhythm by optogenetic stimulation of the DBB/MS complex (Vandecasteele et al., 2014). For that purpose, we infected the LHb with adeno-associated virus (AAV) encoding the fast Channelrhodopsin oChIEF (Lin et al., 2009, Figure 1E). Light stimulation of the DBB/MS robustly induced transitions of the FP from delta to theta state at both the hippocampus and the LHb (Figures 1F,G). Notably, the theta rhythm persisted after light stimulation had ended (Figure 1G), suggesting that the DBB/MS stimulation triggered a network that internally sustained theta rhythm afterward. Indeed, we found no differences in the peak frequency between the tail pinch-induced theta in non-infected animals and light-induced theta in oChIEF-infected animals (Figure 1H), nor in the peak frequency of delta rhythm [Figure 1H, right; repeated measures two-way ANOVA: factor brain state *F*(1,25) = 545.3, *p* < 0.001; factor oChIEF infection *F*(1,25) = 0.779, N.S.; interaction *F*(1,25) = 0.03, N.S.]. Therefore, the two groups showed no difference. Hippocampal and LHb FPs presented high coherence not only in the delta (0.87 ± 0.10, *N* = 15) but also in the theta states (0.88 ± 0.09, *N* = 15, Figure 1I). In addition, the delta–theta transitions from the hippocampus and the LHb were always simultaneous (data not shown).

**FIGURE 2:**
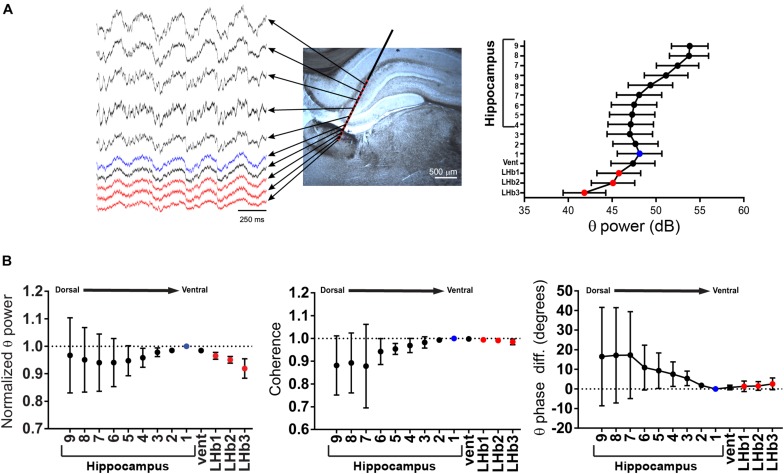
**(A)** Left, schematic representation of an experiment with a silicon probe. Traces are representative recordings during theta from electrodes located at the lateral habenula (LHb) (red dots), from the ventral hippocampal electrode taken as reference (light blue dot, see below), and from the other electrodes (black dots). For clarity, only half of the hippocampal electrodes are displayed. Right, power in the theta band could be readily detected at all recording sites. Data are taken from experiment 2 shank 1 (see Supplementary Figure S1) and presented as mean ± confidence interval. **(B)** Summary of data from four shanks from two experiments. Theta power, coherence, and phase were referred to the recording site with the highest power within the ventral part of the hippocampus (light blue dot). Theta power decreased linearly from the reference recording site onto the LHb (red dots). Coherence was high in all LHb recording sites, and phase difference was small. In contrast, coherence decreased in the dorsal hippocampal recording sites, along with a clear phase shift (vent: presumably ventricle located recording site).

Considering the tight coupling in both frequency and time domains of both field recordings, we decided to study the independence of the hippocampal and the LHb FPs during theta activity. To do that, we performed recordings with linearly arranged silicon probes (2 shanks, with 16 recording sites each, spaced at 100 μm, Figure 2A and Supplementary Figure S1). This allowed us to record simultaneously with a defined geometry from both the hippocampus and the LHb. If active FP generators are present at the LHb, it could be expected that non-monotonic changes of the FP will be detected between the hippocampal and the LHb recording sites. During theta periods, the rhythm was evident at all recording sites (Figure 2A, right; Figure 2B). We thus analyzed theta power, coherence and phase relationship between the recording sites, using as reference a ventral recording site on the hippocampus (Figures 2A,B, see section “Materials and Methods”). There was a small variability of the LHb sites for the three measured variables, and all sites behaved similarly despite their different locations within the LHb (Figure 2B). We found theta power to constantly decrease with distance from the more ventral recordings site on the hippocampus onto the LHb following a linear trend [Figure 2B, left, repeated measures ANOVA: factor recording site *F*(1.066,3.199) = 13.08, *p* = 0.032, test for linear trend after ANOVA: *p* < 0.001]. In addition, we found no significant differences in the theta phase between the LHb and the hippocampus, although the linear trend was still present [Figure 2B, right, repeated measures ANOVA: factor recording site *F*(1.988,5.964) = 1.458, N.S., test for linear trend after ANOVA: *p* = 0.0354]. Finally, coherence values between all the LHb recording sites and the hippocampal reference were close to 1 (0.99 ± 0.004, *N* = 12 from 3 recording sites from 4 shanks and Figure 2B, middle). On the other hand, we found a significant increase in the variability in more dorsal hippocampal recording sites, attributable to the combined effects of variability on the probe pathway and the presence of active generators on dorsal recording sites on the hippocampus (see below).

### Independent Component Analysis Shows No Local Theta Generator at the Lateral Habenula

**FIGURE 3:**
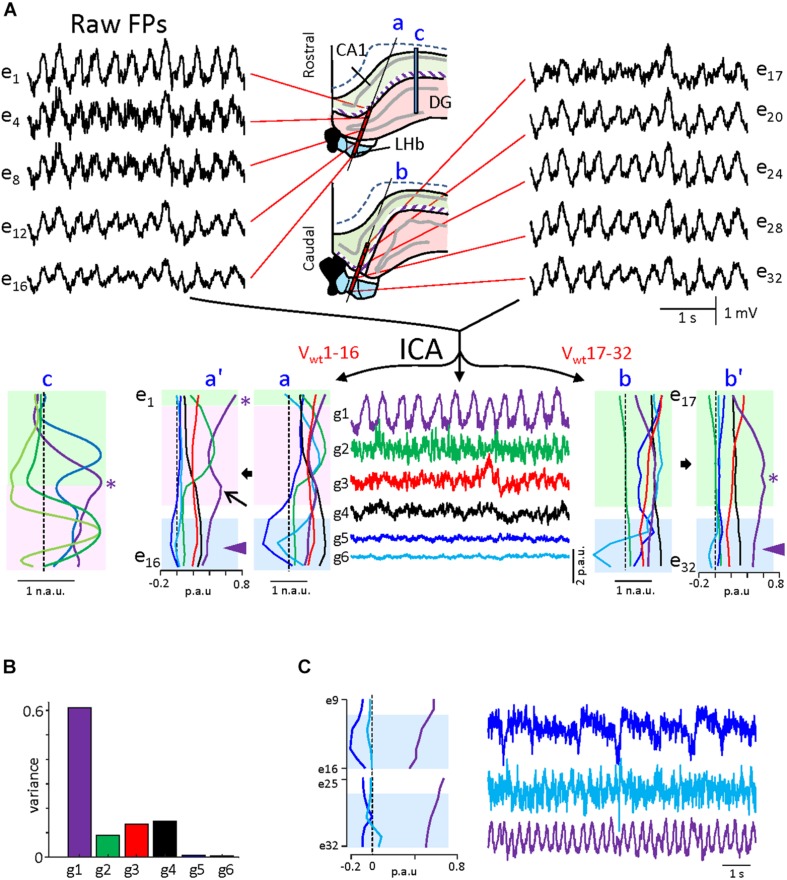
Independent component analysis of linear field potentials (FPs) during theta in experiment 1. **(A)** Sample FPs recorded in the two arrays at the locations indicated in the drawings of the middle panels taken from histological samples. The purple strip marks the position of the apical distal dendrites of the CA1 (st. lac-mol), which is the main source of theta activity in the hippocampus. The middle panel in the lower row shows the time course of the independent component analysis (ICA)-separated generators. The corresponding V-profiles (*V*_*wt*_) are at both sides in normalized **(a,b)** and proportional units **(a’,b’)**. The proportional units provide a quantitative estimation of the relative contribution of each generator on each site. Since some generators are too weak, the normalized curves facilitate visual matching of the voltage gradients to anatomical boundaries. Colored zones denote the different regions spanned by the electrodes. Only *g1* displayed theta activity, and in it presented max power at the st. lac-mol. in all arrays (asterisks). Note that the shape of the V-profiles reflects the relative orientation of the linear array and the anatomical spatial boundaries of the source [compare to the V-profiles obtained from ICA of recordings made orthogonal to the planar portions of cell layers in **(c)**]. The arrow points to a partial maximum in the stratum moleculare of the dentate gyrus (DG), indicating a second source for theta in this region. The theta generator decays smoothly across the habenula (arrowheads), indicating volume conduction from outer sites. The DG generator (g2) contains mostly gamma activity and it peaked in the hilar region because of the granule cell layer folding [note it has no power in array **(b)**]. **(B)** Relative variance contributed by each generator. Those with maxima in the LHb (g5 and g6) were negligible (<1%). **(C)** Reanalysis of the same data for chosen FPs around the LHb regions. The LHb enriched FPs yielded similar generators, while the increased variance makes them more reliable. The g5 displays irregular activity and slow spikes, while g6 shows sporadic theta modulation. The asterisks in **(a–c)** V-profiles mark an equivalent site in the CA1 st. lac-mol.

Since these experiments suggest the absence of FP sources at the LHb, we decided to perform a finer exploration of that possibility. For this purpose, we performed independent component analysis (ICA) of our linear array recordings. ICA is a powerful tool to discriminate the spatial distribution of multiple overlapped FP generators in geometrically defined recordings (Herreras et al., 2015). Since the results obtained from the ICA are sensitive to the stability of the mixture of sources over time (i.e., the electrographic state), we selected theta-only epochs for analysis (see Supplementary Figure S2 for delta-only analysis). Figure 3 illustrates the results obtained for a 30-s period displaying continuous theta. The data matrix was built with the combined recordings from the two shanks of experiment 2 (Supplementary Figure S1), which enables comparing the relative power of the segregated FP components in different regions, hence a better correlation of the voltage profiles (V-profiles) with the anatomical boundaries [we took advantage of the different regions spanned by the two shanks, the rostral one (a) traversing through the dentate gyrus (DG) while the caudal one (b) entering the LHb directly from the CA1 hippocampal region]. As the strong curvatures in this region do not facilitate the identification of the generators by their V-profiles, we included in the comparison the V-profiles obtained from a different experiment in which the linear array (c) traversed orthogonally through a planar section of the CA1 and the DG fields [Figure 3c: from Benito et al. (2016); in the latter, all FP generators are pathway specific and peak in the target structure (CA1 or DG) with or without polarity reversal and decay toward the surrounding structures with a different rate that depends on the subcellular distribution of inputs in the target population (see Benito et al., 2014)]. Such V-profiles are expected to vary when the recording linear array is not orthogonal to the main FP gradients or when the recorded region has strong curvatures, as is the case in the present experiments. Note that the presence of maxima in the V-profile indicates that the sources are contained within the recorded region, while non-zero linear V-profiles indicate distant sources. In the LHb experiments, the ICA returned six FP generators (out of 32 possible) that displayed smooth V-profiles with distinct gradients, albeit only four (g1–g4) exhibited a significant variance (>1%). The spatial distributions (*V*_*wt*_ curves) are presented both in normalized and in proportional units to emphasize either the spatial voltage gradients or the relative power. The strongest generator (g1; >60% of the total variance) was the only one that contained theta (traces in purple), and it peaked in the stratum lacunosum-moleculare (st. lac-mol) of the CA1, decaying toward the DG and the LHb in the rostral shank. The maximum power in the caudal shank appeared toward the middle sites in the array, which matches the place where the array crossed the st. lac-mol of the CA1 after running parallel to the st. radiatum. A small increase in power was observed in the stratum moleculare of the DG (arrow in a’), which indicates the presence of another theta generator in this region, albeit small. An additional hippocampal generator that displayed mostly gamma oscillations peaked in the hilar region (green traces: match to the V-profile in panel c), and it showed negligible activity in the caudal shank that spares the DG. Two other significant generators (red and black traces) exhibited smooth V-profiles in both shanks, indicative of true neuronal sources (in contrast to generators exhibiting noisy V-profiles (Herreras et al., 2015)], but the shape was insufficient to hint on their origin. The remaining two generators (g5, g6) displayed strong voltage gradients within and near the LHb, identifying them as candidates for habenular FP generators. However, both presented a negligible variance (<1%), which opens the possibility of a spurious origin (Herreras et al., 2015). To clarify this, we increased the relative variance of the putative habenular generators by building reduced matrices that contained only the recordings from the deeper half of the electrode arrays. This procedure returned three FP generators. Remarkably, theta was contained in a flat generator (purple trace in Figure 3C), suggesting that it is volume conducted from sources outside the selected recordings, i.e., the hippocampus. Of the other two generators, which resembled g5 and g6, only one reached significant variance (2%), but its temporal pattern was irregular, and no theta oscillations were appreciated (blue trace in Figure 3C). A similar analysis of the two shanks from another experiment confirmed that the LHb is capable of producing some FPs of small magnitude but do not support a local theta generator.

### Pharmacological Inactivation of the LHb Does Not Affect the Recorded Field Potential

**FIGURE 4:**
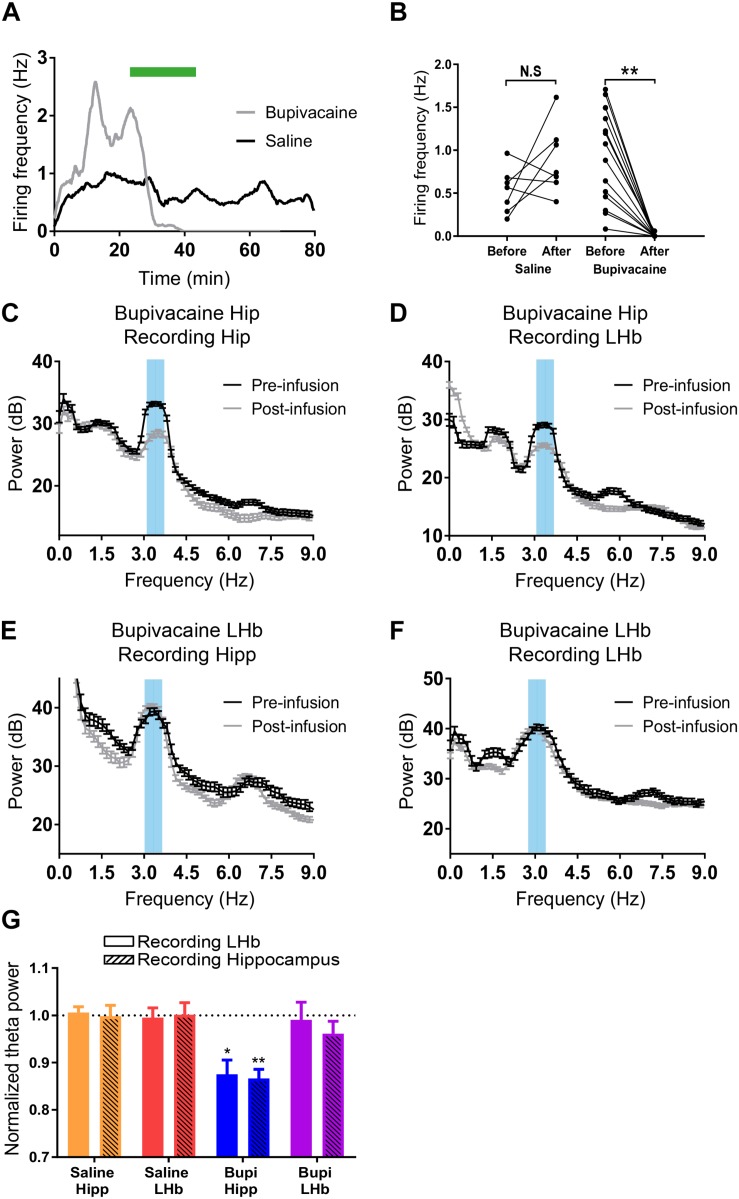
**(A)** Example of the firing rate over time of two hippocampal neurons before, during, and after infusion of saline (black), or bupivacaine (gray). **(B)** Summary of all neurons recorded during saline (*N* = 7, two animals) and bupivacaine infusion (*N* = 15, two animals; ^∗∗^*p* < 0.001). **(C,D)** Example fast Fourier transform (FFT) from a field potential (FP) recording during theta at the hippocampus **(C)** and at the LHb **(D)** before and after bupivacaine injection at the hippocampus. **(E,F)** Example FFT from an FP recording during theta at the hippocampus **(E)** and at the LHb **(F)** before and after bupivacaine injection at the LHb. In all cases, data are presented as power ± confidence interval of the FFT. **(G)** Summary of data presented as theta power after infusion normalized to theta power before infusion. Saline infusion does not affect theta power (hippocampus infusion: 1.06 ± 0.01 and 1.00 ± 0.02 for the hippocampus and the LHb, respectively, *N* = 3; LHb infusion: 0.99 ± 0.02 and 1.00 ± 0.03 for the hippocampus and the LHb, respectively, *N* = 4). Bupivacaine infusion at the hippocampus reduced theta power at both the hippocampus and the LHb (0.87 ± 0.03 and 0.87 ± 0.02 for the hippocampus and the LHb, respectively, *N* = 3; ^∗∗^*p* < 0.01, ^∗^*p* < 0.05 compared to theta power before infusion). In contrast, bupivacaine infusion at the LHb did not affect theta power at either structure (0.99 ± 0.04 and 0.961 ± 0.026 for the hippocampus and the LHb, respectively, *N* = 5).

Evidences presented so far suggest that the FP at the LHb could be interpreted as a volume-conducted signal from a distant generator. To directly test that hypothesis, we attempted to pharmacologically block the LHb or the dorsal hippocampus while recording FP at both structures. To check the efficacy of our approach, we first recorded single neurons while simultaneously infusing by a cannula attached to the recording tetrode at the dorsal hippocampus. Saline infusion at slow rates (0.01 μl/min) did not modify spiking frequency of recorded neurons (Figures 4A,B). On the other hand, infusion of sodium channel blocker bupivacaine at the same rate totally abolished neuronal activity [Figures 4A,B, repeated measures two-way ANOVA: factor before–after infusion: *F*(1,20) = 5.537, *p* = 0.029; factor saline-bupivacaine: *F*(1,20) = 4.038, *p* = 0.058; interaction: *F*(1,20) = 27.55, *p* < 0.001; Sidak’s multiple comparisons *post hoc* test: before–after infusion: *p* = 0.1807 for saline infusion, *p* < 0.0001 for bupivacaine]. This experiment indicates that saline infusion does not affect local activity and confirms blocking of neuronal activity induced by bupivacaine. Thus, we continued to analyze the effect of the infusion on theta power. Saline infusion at either the LHb or the hippocampus did not modify theta power at any of those structures [Figure 4G, repeated measures two-way ANOVA: factor before–after infusion: *F*(1,10) = 0.006, N.S.]. On the other hand, bupivacaine infusion at the hippocampus similarly reduced theta power both at the hippocampus and the LHb [Figures 4C,D,G, repeated measures two-way ANOVA: factor before–after infusion: *F*(1,4) = 60.05, *p* = 0.0015; Sidak’s multiple comparisons *post hoc* test: *p* = 0.0073 for hippocampus and *p* = 0.0164 for LHb; interaction before–after infusion × recording site *F*(1,4) = 0.745, N.S.]. This result confirms that a fraction of the FP recorded in the LHb represents a signal generated at the dorsal hippocampus. To test if locally generated activity could contribute to LHb-recorded FP, we repeated the experiment but now infusing bupivacaine at the LHb. Notably, bupivacaine injection at the LHb did not modify FP theta power at either structure [Figures 4E–G; repeated measures two-way ANOVA: factor before–after infusion: *F*(1,8) = 1.721, *p* = 0.2259], confirming the prediction that contribution of FP generators at the LHb is negligible. Overall above results indicate that most of the FP recordings at the LHb could be attributable to volumetric conduction from other structures.

### Lateral Habenula Neurons Are Coupled With Hippocampal Theta Rhythm

**FIGURE 5:**
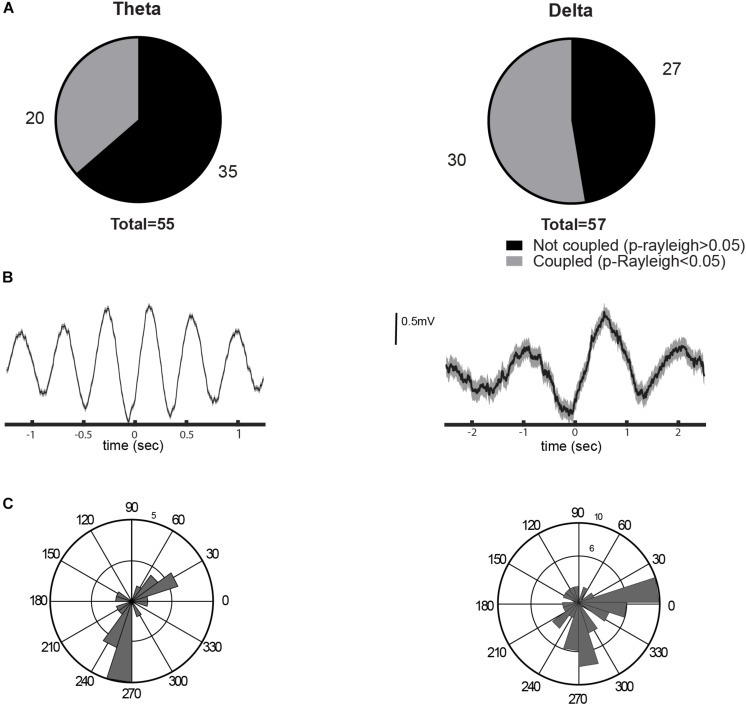
Entrainment of the lateral habenula (LHb) neurons to brain rhythms. **(A)** Proportion of neurons coupled to theta (left) and delta (right) rhythms. **(B)** Examples of spike trigger average of a unit entrained by theta or delta oscillation (left and right, respectively). **(C)** Circular histogram with preferred phases of coupled neurons for each rhythm, to theta on the left and to delta on the right. 0 degree corresponds to the oscillation peak.

Despite the evidence against the biological relevance of FP recordings at the LHb, it has been reported that a fraction of LHb neurons are coupled with hippocampal FP (Aizawa et al., 2013). Therefore, we analyzed the coupling of single LHb neurons to hippocampal theta and delta rhythms. We recorded a total of 55 neurons during theta activity and 57 neurons during delta activity from 15 animals. Of the recorded LHb neurons, 36.6% were coupled to the hippocampal theta rhythm (*p* < 0.05, Rayleigh test for uniformity, Figures 5A,B). Similarly, 52% of the LHb neurons were locked to the delta oscillations (*p* < 0.05, Rayleigh test for uniformity, Figures 5A,B). We recorded 55 neurons in both conditions and, from those, we found 23.6% of neurons to be entrained to both theta and delta oscillations. Theta-coupled cells had two preferred phases, during the ascending and the descending phase of theta wave, while delta-coupled neurons were more concentrated between the ascending and peak of the delta wave (Figure 5C). These results corroborate previous experiments (Aizawa et al., 2013) and indicate that the neurons of the LHb could be entrained to hippocampal theta activity.

### Anatomically Realistic Models Predict That Habenular FPs Are Volume Conducted From the Hippocampus

**FIGURE 6:**
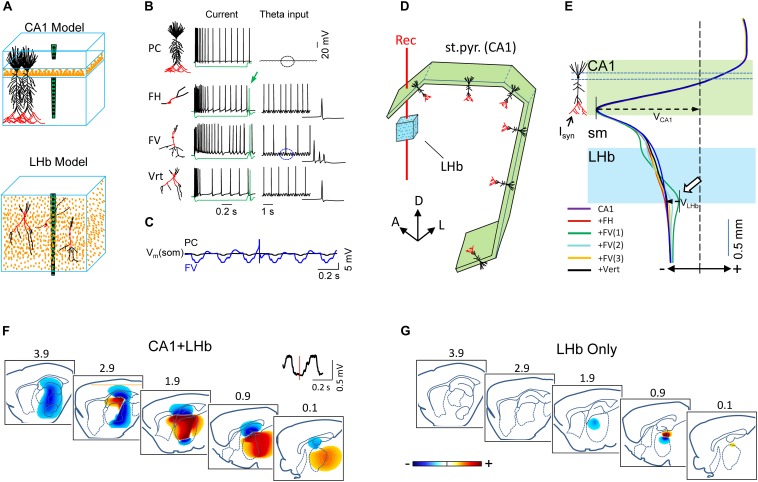
Feed-forward computation of the field potentials (FPs) to explore the origin and volume conduction of theta in a hippocampus/habenula multicellular model of compartmental neurons. **(A)** Each model structure included only the cell types whose partially axialized morphologies make them candidates to generate significant extracellular currents (see section “Materials and Methods”). CA1 pyramidal cell (PC) were arranged in palisade, while habenular neurons were scattered over the tissue, although each class maintained the same orientation. The compartments in red mark the sites where inhibitory synaptic currents were injected at theta frequency on each cell type. **(B)** Model neurons were tuned with V-dependent channels to reproduce the reported firing behavior upon depolarizing current pulses (left column). Habenular neurons fire rebound spikes at the end of hyperpolarizing pulses (green arrow). The right column shows the responses to synaptic theta inputs. Note that the different firing modes shown are not relevant for FP production as the synaptic currents are user guided. FH, FV, Vert: fusiform horizontal, fusiform vertical, and vertical neurons (see section “Materials and Methods”). **(C)** Enlargement of an epoch [marked by ovals in **(B)**] showing the somatic membrane potential (*V*_*m*_) for an PC and a FV cell during theta synaptic input. The theta-modulated *V*_*m*_ is about eight times larger in FV cells because of the smaller size and closer location of inputs to the soma. **(D)** Macroscopic model of the CA1 and LHb structures. The CA1 was built as five blocks of PC units arranged to jointly reproduce the entire septotemporal extent of the CA1 (>300,000 U). Only the position of the st. pyramidale is represented by the green blocks. The red line marks the sites in which computed FPs were selected to mimic an experimental linear recording and to build the V-profiles shown in **(E)**. A, D, L: anterior, lateral, dorsal. **(E)** V-profiles during theta activity generated by distal inputs to CA1 PCs alone (purple) or in combination with some habenular cell types. Three different subcellular distributions of inputs to FV neurons are represented, from +100 μm basal to –200 apical (FV1), from +50 basal to –250 apical (FV2), and from −50 to −400 apical (FV3), and the soma was always free of synaptic input. The CA1 theta V-profile peaked in the st. lac-mol. (*V*_*CA1*_) and decayed smoothly in ventral sites (i.e., volume conduction), traversing the LHb with high amplitude and the same polarity. In most conditions, LHb neurons produced theta potentials (white arrow, measured as *V*_*LHb*_) that were negligible compared to volume-conducted – theta from the hippocampus. **(F,G)** The computed tridimensional voltage produced by the CA1 and/or LHb neurons at an instant of a theta wave marked by the vertical dash in the inset is represented as serial sagittal sections of the brain. Blue/yellow-red colors indicate negative and positive potentials (note that the zones with ± polarity reverse along the theta wave, which in turn is an artificial outcome of AC filtering).

The above experimental results indicate that a fraction of LHb neurons show theta firing modulation, although the extracellular theta recorded in that area appears not to be generated by synaptic currents in the LHb itself. To clarify this apparent paradox, we used feed-forward modeling of FPs and computed the intra (unitary) and extracellular (population) potentials produced by theta-modulated synaptic inputs into realistic CA1 and LHb populations of neurons in a volume conductor representing about one-third of a brain hemisphere (Figure 6; for simplicity, the DG was omitted). We also explored the capability of each structure to produce theta FPs and their spatial extension through volume conduction. Theta-modulated inhibitory synaptic currents were injected into the apical distal dendritic compartments of the CA1 pyramidal cell (PC) population (marked in red in Figure 6A) and/or in three different types of LHb neuron populations [fusiform horizontal (FH), fusiform vertical (FV), and vertical (Vert): see section “Materials and Methods” for details]. The somatodendritic voltage-dependent channels were tuned to produce spike firing adaptation upon depolarizing current pulses (Figure 6B, left column). Coincidently with experimental data, only LHb model cells fired at the end of the hyperpolarizing pulses (green arrow). In addition, LHb cells fired sparsely upon synaptic theta inputs, while PCs generally remained silent during theta epochs (Herreras et al., 1988; Vinogradova, 2001). Note that the firing mode is not strictly relevant for FP generation in this model, since the synaptic inputs are user guided.

The *V*_*m*_ recorded at the soma showed theta oscillations that were much larger in the LHb than in the CA1 neurons (8.4 vs. 1.4 mV, peak to valley: Figure 6C), as expected from their smaller size and shorter distance of inputs to the soma in LHb neurons. However, the somatic *V*_*m*_ is not a reliable correlate of the FPs, which arise mostly from dendritic currents (Elul, 1972; Herreras, 2016), and indeed, the theta FPs produced by the two structures behaved differently. The CA1 model population produced large theta FPs, whereas the LHb populations did not, as it can be appreciated in the V-profiles computed along a recording track traversing both regions (Figures 6D,E). In the CA1, the theta input to distal dendrites produced a dipolar V-profile that peaked in the synaptic zone, reversed polarity below the st. pyramidale, and it decayed at a reduced rate without polarity reversal toward ventral sites, in close agreement with experimental data. Note that the zones with positive or negative polarity reverse along the theta wave, which is, in turn, an artificial outcome of AC filtering (Brankačk et al., 1993; Martín-Vázquez et al., 2013). The volume conduction of this hippocampal theta generator had decayed ∼50% at a distance equivalent to the position of the habenula (light blue zone in Figure 6E, compare to experiments in Figures 3Aa,a’, purple traces). In a broader spatial perspective, provided by the contour plots in selected serial sagittal sections of the brain (Figure 6F), it can be appreciated that the CA1 theta extended over a large brain area and reached maximum amplitude in regions within the concave side of the C-shape structure formed by the CA1 population along the septotemporal axis of the hippocampus. In the animal, this area extends through the habenula, thalamus, and part of the striatum. Regions in the convex side, such as visual, auditory and entorhinal cortices, amygdala, colliculus, and even some mesopontine regions would also receive volume-conducted hippocampal theta of smaller amplitude. It should be noted that the large amplitude of volume-conducted theta in the habenula is build up by currents from all septotemporal levels of the CA1 that form a macroscopic C-shaped dipole. Indeed, it can be shown that removing some parts of it reduced the theta amplitude in the habenula, which explains why theta was reduced but not blocked after the local infusion of bupivacaine in the dorsal hippocampus.

Next, the theta-generating capability of the chosen LHb neuron populations was checked and compared with that of the CA1 population. The FV population turned out to be the most favorable for FP production. However, even in optimal conditions of the subcellular distribution of inputs [traces labeled FV (1–3) in Figure 6E, and contour plots in Figure 6G], the amplitude was 8.2 times smaller than that produced by the CA1 (arrows). The other cell types contributed FPs of negligible amplitude compared to that volume conducted from the hippocampus. Thus, they barely modified the V-profile produced by the CA1 population at the LHb (traces in red or black, Figure 6E). Therefore, we conclude that the cytoarchitecture of the LHb populations of neurons is unfavorable for FP production, and the presence of theta is caused by volume-conducted potentials from the hippocampal generators.

## Discussion

Field potentials are widely used to bring information about neuronal population’s activity. Nevertheless, in many cases, the origin of that signal (i.e., the location of the current generator), and consequently how it should be interpreted, is unclear. In the present work, we aimed at studying the nature of FP recorded at the LHb, particularly theta oscillations. We found considerable evidence suggesting that the theta oscillation recorded at the LHb is mostly a volume-conducted signal from the hippocampus that should not be interpreted as a readout of the LHb local network activity. Thus, we observed a high degree of coherence between the dorsal hippocampus CA1/DG theta and LHb FP with a constant decrease in power from CA1 to LHb and an almost zero phase lag. In addition, ICA showed only very weak generators in the LHb, which mostly exhibit irregular activity. Finally, the inhibition of the neuronal activity at the LHb silenced spiking but did not affect theta power at the LHb or the hippocampus, while the inactivation of CA1 reduced theta power at both structures. Those experimental results were supported by FP modeling that indicated a main contribution of CA1 generated signals to the FP recorded at the LHb.

### The Relevance of Source’s Geometry to Define How Far Theta Waves Are Recorded

The LHb is composed of sparse glutamatergic neurons with very low local connectivity and no laminar disposition of either somata or dendritic arborizations. Consequently, the spatial summation of the extracellular currents at the LHb would be unfavorable for the individual and the collective clustering of currents. Indeed, the ICA showed only very weak generators in the LHb, and they mostly exhibit irregular activity. In a former study (Torres et al., 2019), we experimentally confirmed the theoretical prediction that non-zero linear V-profiles of the ICA separated FP generators correspond to volume-conducted currents from a distant source. Here, we report a main theta generator that peaked in the st. lac-mol of the CA1 and a secondary maximum at the DG. The almost linear decay up to and beyond the LHb thus indicates the volume-conducted nature of FP recorded at those heights.

It should be noted that not all hippocampal generators produce an FP that spreads to the habenula (or anywhere else). In a former study (Fernández-Ruiz and Herreras, 2013), we described that the layered folding of the granule cell population promotes a supernormal clustering of volume-conducted currents generated at the granule cell layer in the concave side (the hilar region), while the outer parts show reduced potentials. Thus, volume conduction is extremely sensitive to the architecture of the source population. In fact, the present model predicts that the C-shaped macroscopic dipole formed by distal CA1 dendrites along the septotemporal axis of the hippocampus produces a supernormal clustering of the theta currents in the encroached regions, including the LHb. A similar explanation has been proposed for the whole-brain recording of slow cortical oscillations contributed by synchronous currents from the spheroid geometry of the cortical mantle (Torres et al., 2019).

The curved architecture of the sources has important implications for the interpretation of results presented in Figures 4, 6. For instance, we found a partial reduction, but not blockade, of theta during the neuronal silencing in the hippocampus produced by the local injection of an anesthetic. Similar findings have been reported in the literature for decades, which lead to wrong interpretations of the mechanisms and the participation of different brain structures in the development of FP patterns (Herreras et al., 2015). Our macroscopic modeling of FPs predicts that even extensive chemical blockade of the hippocampus would affect only a small fraction of such extended sources, since large parts of it still promote strong volume-conducted potentials due to their curved structure. This phenomenon could be detected even in regions that have been totally devoid of local activity. For instance, large-amplitude theta activity can be readily recorded in the silenced visual cortex, and slow cortical oscillations receive significant contributions from distant cortical regions (Torres et al., 2019).

Weiss and Veh (2011) reported that neurons of the LHb could be morphologically classified in six types, three of which have multipolar morphology that shall produce short-distance cancelation of inward and outward transmembrane currents (close fields). In contrast, the other three morphologic neuronal types present a certain degree of axialization, which would allow the generation of unitary dipoles and have been modeled here. Nevertheless, their scattered distribution in the tissue shall lead to mesoscopic cancelation by the volume average of different cells and cell elements, as reported for structures similarly unorganized as the lateral septum (Martín-Vázquez et al., 2016). A common orientation of such neurons in the volume may, however, lead to mesoscopic fields that reach significant amplitude, as we find here for the FV neuron type when inputs were restricted to an optimal dendritic band and theta-modulated currents were modeled. However, we find no such small theta generator in experiments in the LHb. We thus speculate that those neurons do not receive theta input, while other multipolar classes of habenular cells may. Further experiments are needed to clarify the local circuitry and the topology of extrinsic inputs in the LHb.

### Functional Connectivity of the LHb Based on FPs or Spikes

In addition to FP recordings, some articles studied theta modulation of LHb neurons firing (Aizawa et al., 2013; Baker et al., 2015; Yang et al., 2018). Particularly, Aizawa et al. (2013) found a fraction of LHb neurons to fire coupled to hippocampal theta. Our results are in agreement with and confirm those results. Thus, we find that about half of the neurons have a theta phase preference. Therefore, the general hypothesis that LHb is entrained to ongoing theta rhythm would be sustained by our results.

The LHb provides information about objective and subjective value of stimuli used to guide behavior. Several evidences indicate that the LHb signaling is relevant for hippocampus-dependent learning (Lecourtier et al., 2004; Tomaiuolo et al., 2014; Chan et al., 2017). The mechanisms by which the LHb and the hippocampus could interchange information are not obvious since those structures are not directly connected. Previous articles suggested that the LHb influences the hippocampus through a Raphe-mediated loop (Ferraro et al., 1997). In addition, the hippocampus and the LHb receive common inputs from forebrain structures such as DBB/MS, which constitutes a theta rhythm generator (Petsche et al., 1962). Thus, the interaction between both structures could be guided by common inputs from DBB/MS that would synchronize them in theta rhythm. Such hypothesis was analyzed by previous articles (Aizawa et al., 2013; Goutagny et al., 2013; Baker et al., 2015; Yang et al., 2018). Some of them provided information indicating that the LHb and the hippocampus could synchronize their activity at theta frequency (Aizawa et al., 2013; Goutagny et al., 2013). The synchronization was expressed as coherence of the FPs recorded in both structures (Goutagny et al., 2013) or by phase coupling between single neuron activity at the LHb and hippocampal theta (Aizawa et al., 2013). In addition, it was shown that theta rhythm recorded at the LHb correlates with animal running speed and reward approach, providing a potential biological relevance to that signal (Baker et al., 2015). In view of our results, conclusions from some of those articles should be revisited. Thus, if there is a common input reaching two different structures and this could plausibly indicate that they belong to functionally related circuitry processing similar information, the use of FPs as supporting evidence may be misguiding unless the actual origin of the FP source is identified. It is not infrequent to find reports in the literature in which some units show lockstep firing to FP oscillations or patterns in some states or behavioral tasks but not in others (Donoghue et al., 1998; Canolty et al., 2012). Since FPs are mostly made up of synaptic currents, such cases cast doubts as to the correct identification of the site where these currents were originated. Here, we show with the biophysical model that theta input to different LHb cell types may indeed produce theta modulation of their spike output, while the contribution of such theta currents to extracellular FPs depends on the specific portion of the dendritic tree where synapses are activated and other architectonic factors, and in most cases, it is negligible.

### Concluding Remarks

In recent years, the use of advanced spatial techniques is reporting a growing number of well-known FP patterns and oscillations whose origin turns out to be away from recorded sites (Fernández-Ruiz et al., 2013; Carmichael et al., 2017; Marmor et al., 2017; Parabucki and Lampl, 2017; Torres et al., 2019). We expanded the list by including the non-local nature of theta oscillation of the FP at the LHb. Our results are an example of the need to deeply investigate complex biological signals such as the FP before arriving to conclusions.

Our model was based on limited information and should be considered only a first step into the analysis of FPs at the LHb. It indicates that only optimal subcellular distribution of DBB/MS inputs onto optimally distributed FV cells would lead to a local generated FP in the LHb. Since neither dendritic distribution of DBB/MS inputs nor spatial distribution of FV cells in the LHb have been studied, we could not discard that possibility. However, we found no experimental evidence of locally generated FP in the LHb for either theta or delta oscillation. The existence of a locally generated FP in higher frequencies (e.g., gamma rhythm, Goutagny et al., 2013) remains to be investigated.

We confirmed theta modulation of the firing of LHb neurons. Presumably, that modulation originates from septal/DBB inputs to the LHb; however, this hypothesis has not been directly tested. Furthermore, the relevance of theta entraining of the LHb neurons has not been investigated, neither it is understood if and how information from the LHb reaches and exerts influence over the hippocampus. Future experiments should be done analyzing all these points.

## Materials and Methods

### Animals

Recordings were performed in Wistar male rats weighing ∼300 g. Rats were obtained from the School of Biochemistry, University of Buenos Aires, and were housed under a 12-h light/12-h dark cycle (lights on at 8 AM), with food and water available *ad libitum*. All procedures were in accordance with local institutional regulations on the use of laboratory animals (IACUC of the School of Medicine, University of Buenos Aires, 0052635/2017).

### Virus Injection

Virus infusions were made on Wistar male rats (∼250 g). Animals were anesthetized with isoflurane (1.2% with a 1.25 l/m O_2_ flux) and mounted in a stereotaxic frame (Stoelting Co., Wood Dale, IL, United States). To infuse the virus in the DBB/MS complex, a small craniotomy was made (AP, +1.1; ML, +2.8; DV, −7.2, 20°; Paxinos and Watson, 2007). A 2/8 AAV-Syn-oChIEF-tdTomato virus (Nabavi et al., 2014) was injected through a stainless-steel tube cannula connected to a microsyringe (ILS, Germany) controlled by an infusion pump (MD-1000 and MD-1001, Bioanalytical System, United States). One microliter was injected at a 0.1-ml/min rate. The cannula was left at the final location for 10 min, and then, it was slowly withdrawn to avoid reflux. At the end of the surgery, animals were injected with a single dose of meloxicam (0.2 mg/kg) as analgesic and gentamicin (2.5 mg/kg) as antibiotic. After the surgery, animals were left undisturbed for recovery for 3 weeks until recordings were made.

### Electrophysiological Recordings

*In vivo* electrophysiological recordings were performed under urethane anesthesia (1.6 g/kg, i.p.). Local anesthetic (bupivacaine 5 mg/ml) was applied to pressure and incision points. Animals were secured to a stereotactic frame (Stoelting Co., United States); temperature was controlled with a servo-controlled heating pad (37°C ± 0.5; Fine Science Tools, Vancouver, Canada). Additional doses of urethane were applied to maintain a constant level of anesthesia, periodically determined by hind-limb reflexes, whisker movements, and brain activity pattern.

Dorsal hippocampal recordings were done with tetrodes (tungsten, 50 μm, California Fine Wire, United States, ∼50 kΩ impedance) with their tips spaced ∼200 μm (AP, −3.6; ML, −2.6; DV, −2.8; Paxinos and Watson, 2007). For the LHb recordings, an arrangement of three tetrodes (four tungsten 20 μm twisted wires, California Fine Wire, United States ∼100–200 kΩ impedance) glued together were used (AP, −3; ML, −2.2; DV, −5.3, 20° (Paxinos and Watson, 2007). In some experiments, recordings were performed with a linear silicon probe (NeuroNexus Technologies, 2 shanks, 16 recording sites with 100 μm vertical spacing, A2 × 16–10 mm-100-500-177). A screw located in the middle line above the cerebellum was used as reference, and the animal was grounded through the stereotaxic frame. Electrophysiological data was amplified (×400), filtered (0.3–10 kHz) and sampled at 25 kHz employing an RHA2116 (Intan Technologies, LLC, United States) or an Amplipex system amplifier (KJE-1001, Amplipex LTD., Hungary).

An optic fiber (200 μm y NA of 0.37; Thorlabs) was targeted to the same coordinates where the virus was injected. Laser intensity was adjusted to 20 mW measured at the end of the optical fiber. We used pulses of 10 ms at 15 Hz to induce delta to theta state transitions.

### Pharmacological Manipulations

To silence neuronal activity, bupivacaine (5 mg/ml), a voltage-gated sodium channels blocker, or sterile saline solution (control experiments) were infused. In experiments aimed at recording single-unit activity, we infused 0.2 μl at a rate of 0.01 μl/min. In experiments where only FP was recorded, we infused 1 μl at or 0.1 μl/min. The solution was injected with a 10-μl syringe (ILS, Germany) attached to a polyimide tube (200 μm diameter) and the rate controlled with an infusion pump (MD-1000 and MD-1001, Bioanalytical System, United States). Recordings at the site of infusion were performed by the same three tetrodes’ array described above glued to the polyimide tube.

### Data Processing and Analyses

The raw signal was resampled to 1,250 Hz to be used as an FP signal. Spike detection and automatic sorting were performed using Klusta and refined manually using Kwik-gui (Rossant et al., 2016). All data were analyzed by custom-made scripts or the Chronux package (“Observed Brain Dynamics”)^1^. Spectral analysis was performed using multitaper estimates with a window length of 2 s and a 50% overlap over frequencies ranging from 0 to 10 Hz.

After the visual inspection of the signal and the power spectrum, segments of FP were classified as delta or theta. Both a clear band in the power spectrum (centered at ∼0.9 Hz for delta and ∼3.5 Hz for theta) and a clear signal was necessary to include a segment in any category.

Phase locking of each unit to different rhythms was analyzed by Rayleigh test for uniformity of FP phases of unit firing. Units were considered to be phase locked when they displayed a *p* < 0.05. For that analysis, we used FP recorded at hippocampal CA1. FP’s instantaneous phases were obtained from the Hilbert transformation of each filtered signal.

### Silicon Probe Recordings

Coherence phase and power were calculated in reference to the recording site at the ventral aspect of the dorsal hippocampus with the highest theta power. In two shanks, that reference corresponded to the most ventral recording site, whereas in two other shanks, it corresponded to the second most ventral recording site. In the latter, the most ventral hippocampal recording site was excluded from the pooled analysis performed in Figure 2B. In addition, in Figure 2B, we limited our analysis to the minimum number of electrodes obtained in all experiments (three at the LHb and nine at the hippocampus, beginning from the one selected as reference).

### Statistical Analysis

Statistical analysis was performed using Graph Prism (GraphPad Software). Statistical test used in each case is described in section “Results”.

### Histology

Fiber optic and electrodes were dyed with DiI (Thermo-Fisher, United States) to facilitate the anatomical reconstruction of the recording sites. After each experiment, animals were deeply anesthetized and decapitated. The brain was removed and placed in a 4% paraformaldehyde in phosphate-buffered saline solution. The localization of the electrodes and fiber optics was determined by transmitted light and red fluorescence microscopy.

The position of the recording site in silicon probe electrodes was calculated as follows: Dil staining was used to determine the ventral tip of the probe at the LHb. From the ventral tip, we determined the position of recording sites within the LHb. The recording site immediately dorsal to LHb was considered to be located in the ventricle. Following dorsal recording sites were considered to be in the hippocampus and labeled with an increasing index from ventral to dorsal.

### Independent Component Analysis

The ICA is a blind source separation technique that separates the components blended in compound signals such as the FPs. The performance and interpretation of the separated components largely depends on the nature and the characteristics of the signals (Bell and Sejnowski, 1995; Stone et al., 2002). It is routinely used to elucidate the different neural sources contributing to electroencephalography recordings at the scalp or in functional MRI (Onton et al., 2006; Rogers et al., 2019). The application of an ICA to intracranial FPs (Makarov et al., 2010) is much more informative, since linear multielectrode arrays may be positioned to span the volume occupied by the sources themselves. Indeed, they can even resolve the voltage shells produced by different synaptic inputs arriving at the same cell population (so-called FP-generators), as long as they do not completely overlap in the same dendritic territory (Benito et al., 2014). We chose algorithms that optimize spatial coherence in the segregated components, which complies with the principle of charge conservation and the instantaneous character of the electrical fields. The kernel density ICA algorithm (Chen, 2006) was employed, and customarily implemented in Matlab. Recorded FP signals *u*_*m*_(*t*) were modeled as the weighted sum of the activities of *N* neuronal sources or FP generators:

$$u_{m}\,\left(t\right)=\sum_{n=1}^{N}V_{m\,n}\,s_{n}\,\left(t\right),m=1,2,\ldots ,M$$

where (*V*_*mn*_) is the mixing matrix composed of the so-called voltage weights (*V*_*wt*_) of *N* FP generators on *M* electrodes, and *s*_*n*_(*t*) is the time course of the *n*th FP generator. Thus, the raw FP observed at the *m*th electrode tip is a linear mixture of the electrical activity of several independent FP generators. Using *u*_*m*_(*t*), the ICA finds both (*V*_*mn*_) and *s*_*n*_(*t*).

Before performing the ICA, FPs were preprocessed through dimension reduction using the principal component analysis, which efficiently diminishes the presence of noisy weak generators (Makarova et al., 2011). We routinely rejected noisy components with a total variance below 1% (i.e., always keeping 99% of the original FP variance in the matrix), unless their spatial and temporal accuracy could be ensured by other means. The mathematical validation and practical limitations of this approach, as well as the possible causes of faulty separation, have been thoroughly investigated using realistic modeling (Makarova et al., 2011, 2014).

The joint curve of *V*_*wt*_ of an FP generator reflects the instant depth profiles of the proportional voltage among sites, which are analogous to the depth profiles of the evoked activity (Korovaichuk et al., 2010). This information together with anatomical data can be used to find the location, polarity, and additional geometrical features of the source (see Herreras et al., 2015 for restrictions). Thus, linear or curved V-profiles of the components indicate that the sources are located in sites remote to the electrodes (FPs arrive to all electrodes through volume conduction with similar power) or that they are nearby (strong voltage gradients develop inside and close to the sources), respectively (Herreras et al., 2015; Torres et al., 2019). Such profiles are accurate to the subcellular level in monolayered structures, while they are less informative in glomerular structures in which they reproduce the joint field distribution of the activated populations (Makarova et al., 2014). For the current purposes, it is important to accurately match the V-profiles to anatomical boundaries, always keeping in mind the effects of curvatures and the relative orientation of the recording shanks.

The performance of the ICA may differ somewhat depending on the temporal structure of the FPs and the degree of spatial overlap of the sources. Spatial and temporal features of the signals deserve consideration. For the present purpose of elucidating whether theta is produced in both the hippocampus and the habenular complex, we must consider the possibility of a common synaptic input from the DBB/MS neurons to both structures. Although common inputs to separate targets do not warrant local generation of FPs in all targets (e.g., Martín-Vázquez et al., 2013), in case they do, the expected high coherence between them will prevent ICA to separate them in two different components. However, we can take advantage of the large extent spanned by linear arrays to examine the V-profiles obtained simultaneously in both structures. In former studies, we found that structure-selective FP generators have a V-profile with local maximum and linear decay in adjacent structures, while coherent activity in two structures returns FP generators with maxima in both (Benito et al., 2014, 2016). We should also consider the markedly different electrographic patterns displayed by the hippocampus (theta/irregular activity), which reflects a different composition of the synaptic inputs generating the raw FPs. Ideally, the statistical properties of the mixture should remain constant, else the ICA may return unstable, duplicated, and hybrid generators (Makarova et al., 2011; Herreras et al., 2015). To minimize this problem, we analyzed epochs with a homogeneous electrographic state.

### Power of FP Generators

The time evolution of the power of a FP generator (in mV^2^) was calculated by:

$$P\,\left(t\right)=\int H\,\left(t-\tau \right)\,v^{2}\,\left(\tau \right)\,d\tau ,$$

$$H\,\left(x\right)=\left\{ \begin{array}{c} 1/\Delta \;\text{if}\,x\in \left[-\Delta /2,\Delta /2\right]\\ 0,\text{otherwise} \end{array}\right.$$

where *v*(*t*) is the virtual FP at the electrode with maximal power, and Δ is the length of averaging. The overall mean power is then defined by setting Δ equal to the complete time interval. Statistical analyses and data treatment were performed in MATLAB^®^ using the Statistics and Machine Learning Toolbox. The ICA was performed using the LFPsource^®^ software running in MATLAB environment and freely available at: http://www.mat.ucm.es/∼vmakarov/downloads.php.

### Forward Model of Field Potentials

#### General Design

Forward models allow the 3D voltage shell to be estimated near and far from the activated population. A multicellular aggregate of compartmental units of realistic morphology endowed with Hodgkin–Huxley dynamics was used (Lindén et al., 2011; Makarova et al., 2011; Torres et al., 2019). Such models allow synthesizing FPs from controlled mixtures of synaptic inputs to specific subcellular domains that interact naturally within cells (Makarova et al., 2011), whereas the extracellular part of transmembrane currents add linearly in the simulated volume (model FPs). In this approach, the input consists of a series of instants when synaptic channels are activated, and the temporal envelope of the transmembrane currents is computed from channel kinetics.

Field potentials in a volume equivalent to about one-third of a brain hemisphere were computed. The corresponding boundaries in the rat are as follows: from midline to 4 mm lateral; from the septal complex to the subiculum (4 mm) in the anteroposterior axis; and the entire dorso-ventral extension. The calculations were made for independent blocks of tissue made with a realistic 3D cytoarchitecture that jointly formed the two structures of interest, the hippocampal CA1 and the habenular complex, which were at realistic distances and orientation from each other. Since the experiments indicated that the CA1 region is the only significant theta generator in the hippocampus under urethane anesthesia (see also Stewart and Fox, 1989), the DG was not modeled in this study. in addition, we only modeled the cell types that present an appropriate morphology to contribute to FPs in each structure (Lindén et al., 2011; Herreras et al., 2015), which were the PCs in the CA1, and three subtypes of habenular neurons that exhibit a certain degree of axialized dendritic structure (Weiss and Veh, 2011). These were the FH, FV, and Vert neuron classes. Polymorphic, spherical, and neurogliaform cells were excluded as sources of extracellular currents.

Field potentials were estimated by distance-weighted addition of all the compartmental currents obtained in each neuron model for each of the regions. The activation of a block of tissue was achieved by replicating the currents through a system of spatial coordinates that represented the compartments of all neurons (Makarova et al., 2011; Martín-Vázquez et al., 2016). Thus, modeled neurons were not synaptically connected, and the dynamics of activation were established by the user as predefined sequences of afferent spikes activating synaptic currents in dendritic compartments that together produced theta temporal envelopes akin to the experiments. The transmembrane currents were calculated using the GENESIS simulator applying an exponential Euler method (Bower and Beeman, 2003).

To describe the macroscopic reach of the FPs, we used a cubic grid of 100 mm to establish the spatial points where the transmembrane currents were integrated. The grids were large enough to collect the parts of the 3D voltage shell required for experimental comparison, and they are represented in five sagittal sections of the rat’s brain separated by 1 mm. Spatial contour plots of the FPs were built at a specific time instant (snapshots). In addition, linear profiles were represented in the center of the populations to enable comparison with the linear spatial profiles obtained in experiments. The conductivity of the tissue was set as 0.33 S/m, as reported *in vivo* (López-Aguado et al., 2001), and the calculations of the FPs were program in MATLAB. In this model, we did not include the effects of tissue heterogeneity and anisotropy. In a former study (Torres et al., 2019), we found that the magnitude of possible errors is only significant near to regions of high tissue resistivity.

#### The CA1 Model

The CA1 was made as five blocks of tissue with a constant width of 2 mm and a cell density of 54 U in a 50 × 50-μm lattice (352,756 CA1 PC units arranged in a palisade-like manner). The blocks jointly extended through 10.2 mm, and they were assembled at certain angles into a C-like structure that reproduced the curved septotemporal axis. The assembly was tilted 45° over the sagittal plane (temporal end out) to account for the mediolateral displacement. For simplicity, the rotation over the anteroposterior axis was not implemented. The morphology, electrotonic parameters, and the subcellular distribution of active channels in the PC neuron model were as reported earlier (Varona et al., 2000; Makarova et al., 2011). The model included 13 types of ion channels with an optimized subcellular distribution to simulate active somatodendritic properties (Johnston, 1996; Ibarz et al., 2006; Makarova et al., 2011). In the subthreshold regime of synaptic inputs explored here, there was only a minor contribution of active properties to CA1 FPs (Martín-Vázquez et al., 2013, 2016). Theta activity was simulated as a single inhibitory input (Unal et al., 2015; Salib et al., 2019) to the distal apical dendrites that reproduces well the spatial profiles in anesthetized preparations (Buzsáki et al., 1986; Brankačk et al., 1993), displaying a maximum toward the hippocampal fissure and a polarity reversal in the stratum radiatum near the cell body layer. Synaptic inputs of the GABA-A type (*t* = 30 ms) were used in theta sequences to mimic rhythmic activation, and we employed synaptic conductances of 24–28 nS. Note that the chemical nature of the synaptic current (excitatory or inhibitory) is scarcely relevant to achieve near sinusoidal FPs, since anyone renders similar spatiotemporal FP envelopes in AC-coupled recordings (Martín-Vázquez et al., 2013).

#### The Habenular Model

We simulated theta-paced inputs from the DBB/MS complex to the LHb (Aizawa et al., 2013). Since some data relevant for an accurate model of FPs produced by LHb neurons are lacking, we approached those that we found earlier to be less relevant or that become averaged when scaled up to a population scale (e.g., cell orientation, fanning angle of dendritic branches, intrinsic currents). A block of 0.7 × 0.7 × 0.7 mm of tissue was set, which contained three types of LHb model neurons (FH, FV, and Vert) distributed uniformly in the following proportions (Geisler et al., 2003; Weiss and Veh, 2011): FH, 19% (10.580 U); FV, 6.5% (3,600 U); Vert, 5% (2,700 U). The neurons’ morphology was built with 35, 51, and 87 compartments, respectively, plus 35 additional compartments for the axon that was only used to check for cell firing properties (i.e., not included for FP estimation).

The neuron models were endowed with active dendritic properties. According to the available literature, the firing patterns found in the LHb neurons keep no relation to specific morphological types (Weiss and Veh, 2011; Aizawa et al., 2013). Thus, we fine-tuned a somatodendritic channel repertoire for each neuron class that reproduced such firing patterns. Since the output of model neurons is not fed into other habenular neurons, the firing patterns were not relevant for FP production, and thus, they were included to assess electrophysiological performance.

Necessary data relative to the specific neuron target of theta inputs and their subcellular coverage are also lacking. Hence, we scrutinized putative theta-paced inputs into the neuron classes whose morphology is favorable to produce significant FPs (FV, FH, Vert). It should then be understood that inputs to other cell types are not expected to contribute significantly to FPs, as they produce closed fields (Lorente de No, 1947; Herreras et al., 2015). In addition, we limited synaptic activation to portions of the dendritic trees (compartments placed at ± 100–200 μm from the soma) to enable the generation of dipoles by individual cells. Again, if theta inputs homogeneously cover the dendritic trees, massive cancelation of randomly oriented elementary dipoles would minimize the chances to scale up in the volume and produce significant FPs (Martín-Vázquez et al., 2016). Finally, specific data on the chemical nature of the DBB/MS input to LHb neurons are also lacking, and we used the same GABAA-mediated activation pattern as for CA1 PCs.

## Data Availability Statement

The datasets generated for this study are available on request to the corresponding author.

## Ethics Statement

The animal study was reviewed and approved by IACUC of the School of Medicine, University of Buenos Aires, 0052635/2017.

## Author Contributions

NB-C performed the experiments, analyzed the data, and wrote the manuscript. JM and OH analyzed the data, performed the modeling, and wrote the manuscript. AM performed the experiments that started the project. DG-V designed the software. RS-P designed the project. MB and JP designed the project and wrote the manuscript.

## Conflict of Interest

The authors declare that the research was conducted in the absence of any commercial or financial relationships that could be construed as a potential conflict of interest.

## Footnotes

1 http://chronux.org
