# Supplementary material for: Volume-Conducted Origin of the Field Potential at the Lateral Habenula
Source: Front Syst Neurosci. 2020 Jan 8;13:78. doi: 10.3389/fnsys.2019.00078 (PMC6961596; doi:10.3389/fnsys.2019.00078)

Supplementary Figure 1. Photomicrograph of electrode location at the LHb and at the Hippocampus of the experiments performed with silicon probes. Electrodes were labelled with DiI to visualize their position by red fluorescence. Pictures are images from transmitted light (upper left), red fluorescence (upper right) and a merged of both (lower). Each plate corresponds to a different experiment, electrode-shank and anatomical structure. Red dots represent the inferred location of recording sites at the LHb.

Experiment 1
Shank 1

LHb

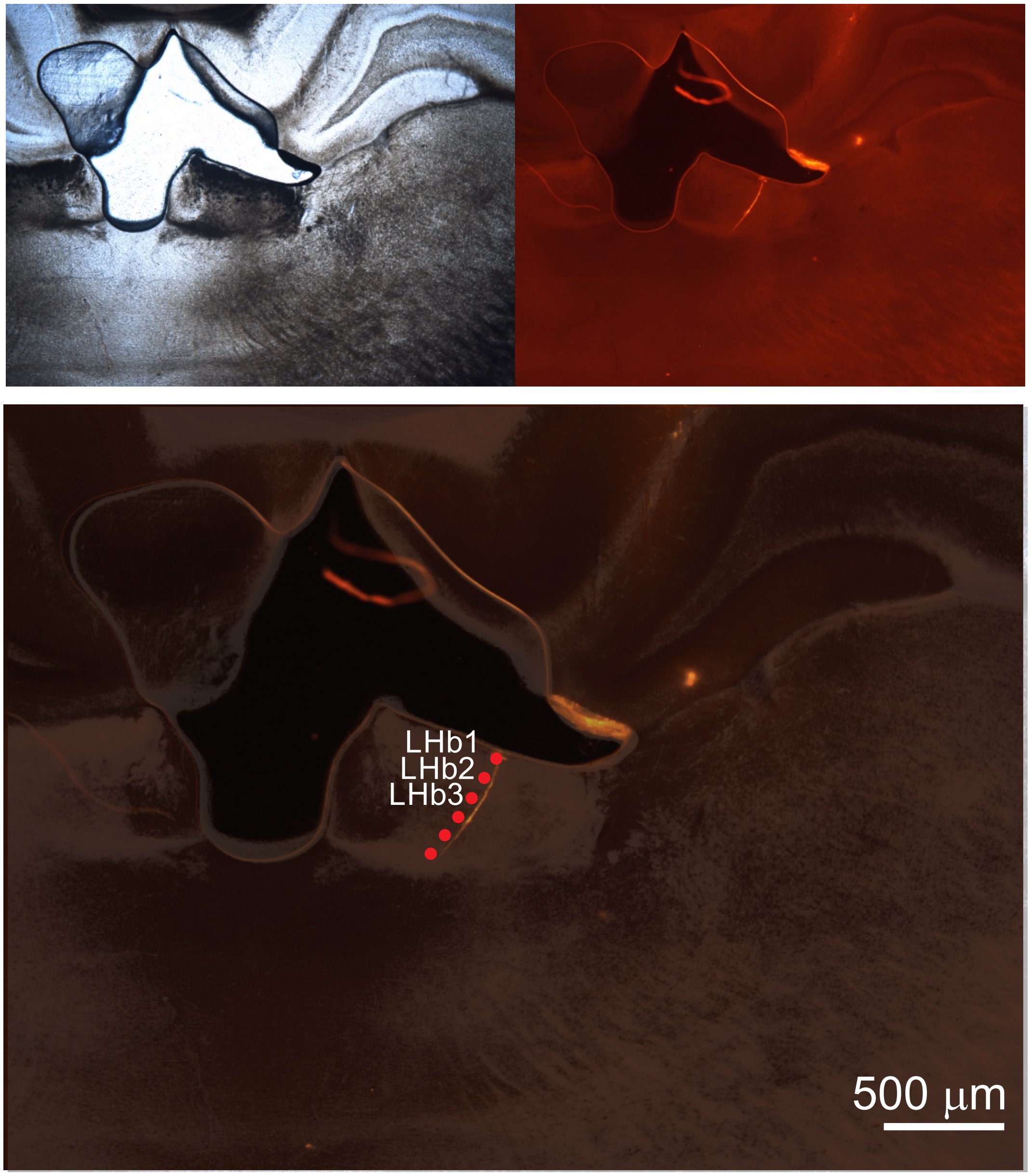

Experiment 1
Shank 1

Hippocampus

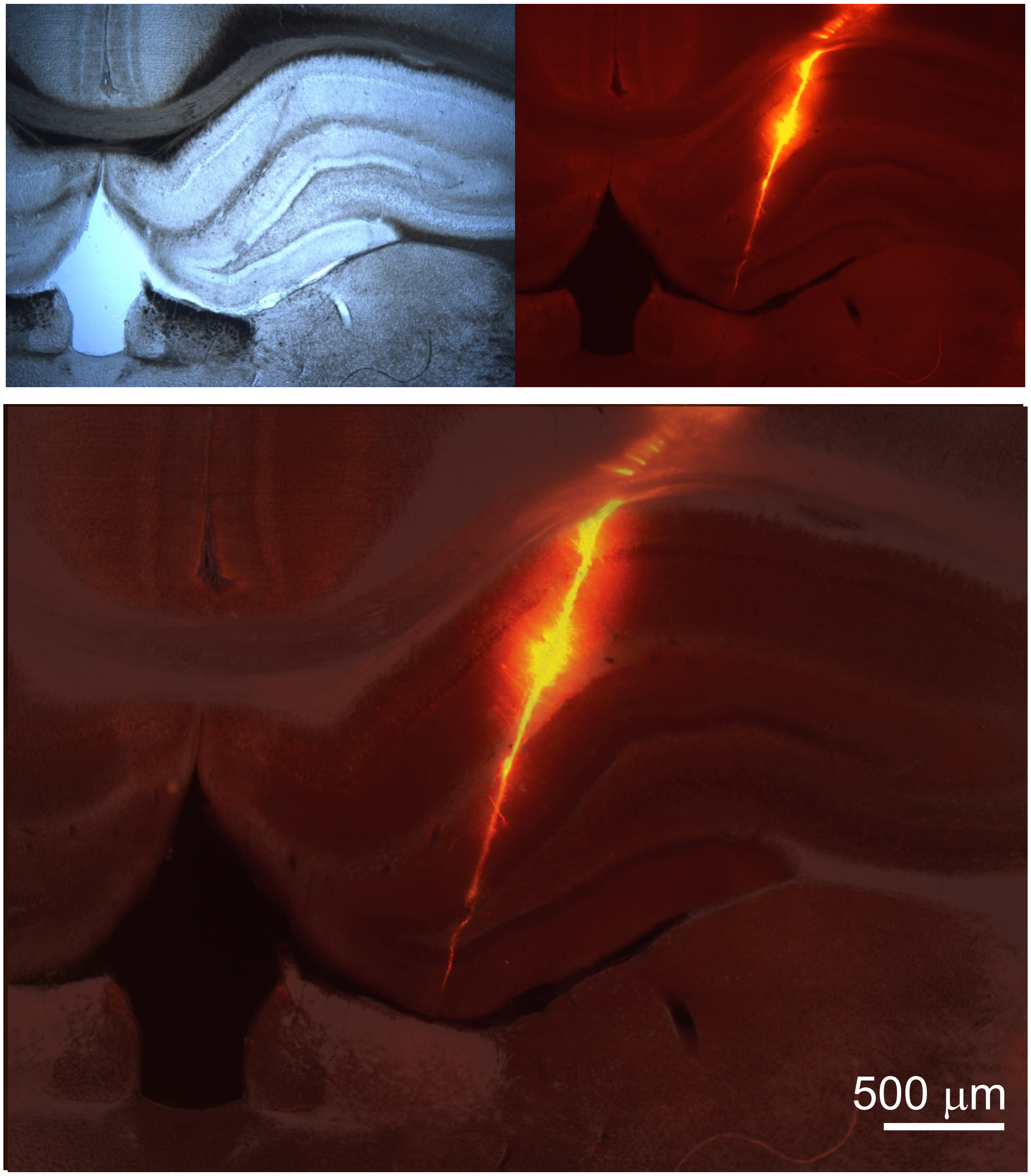

Experiment 1
Shank 2

LHb

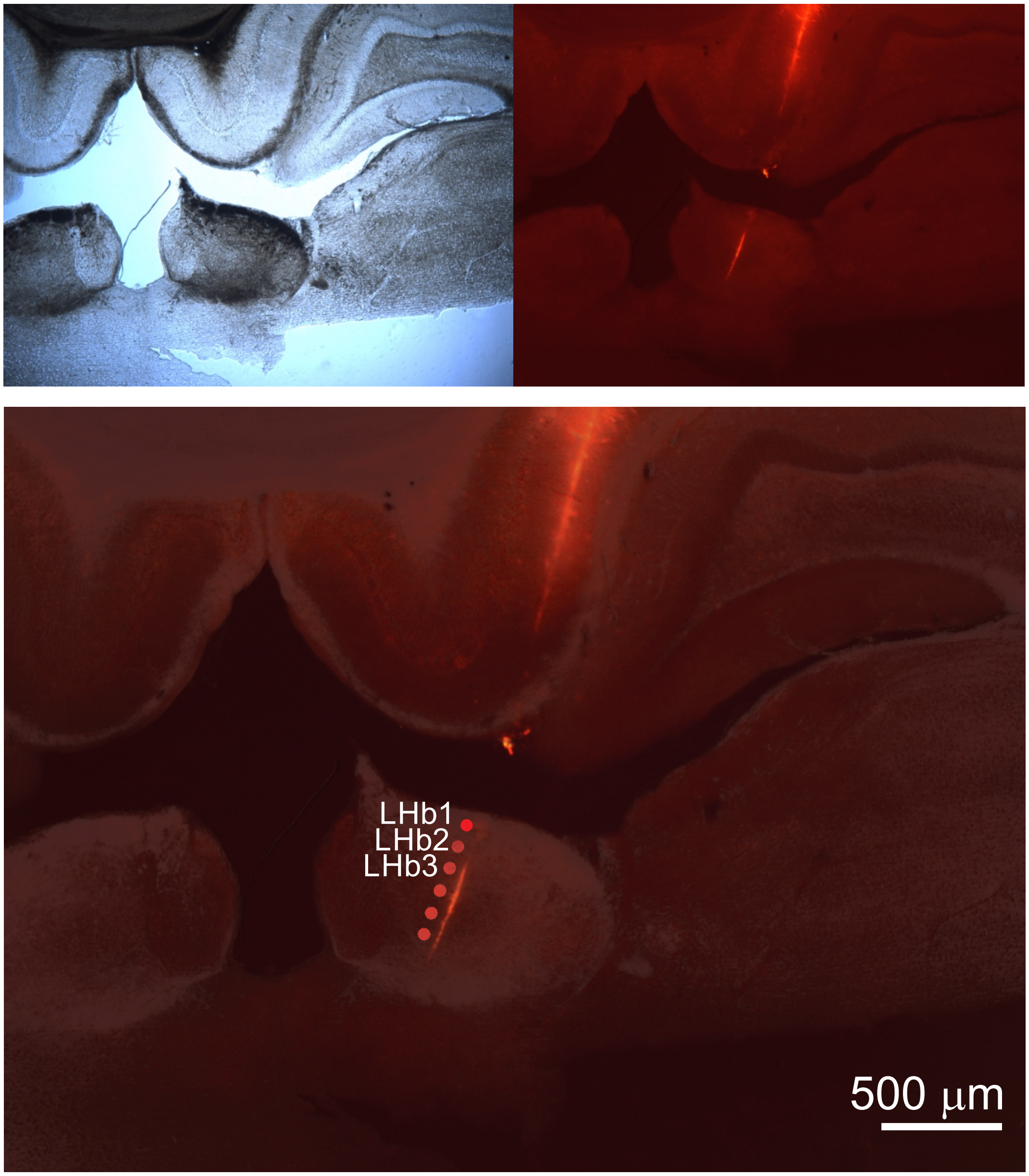

Experiment 1
Shank 2

Hippocampus

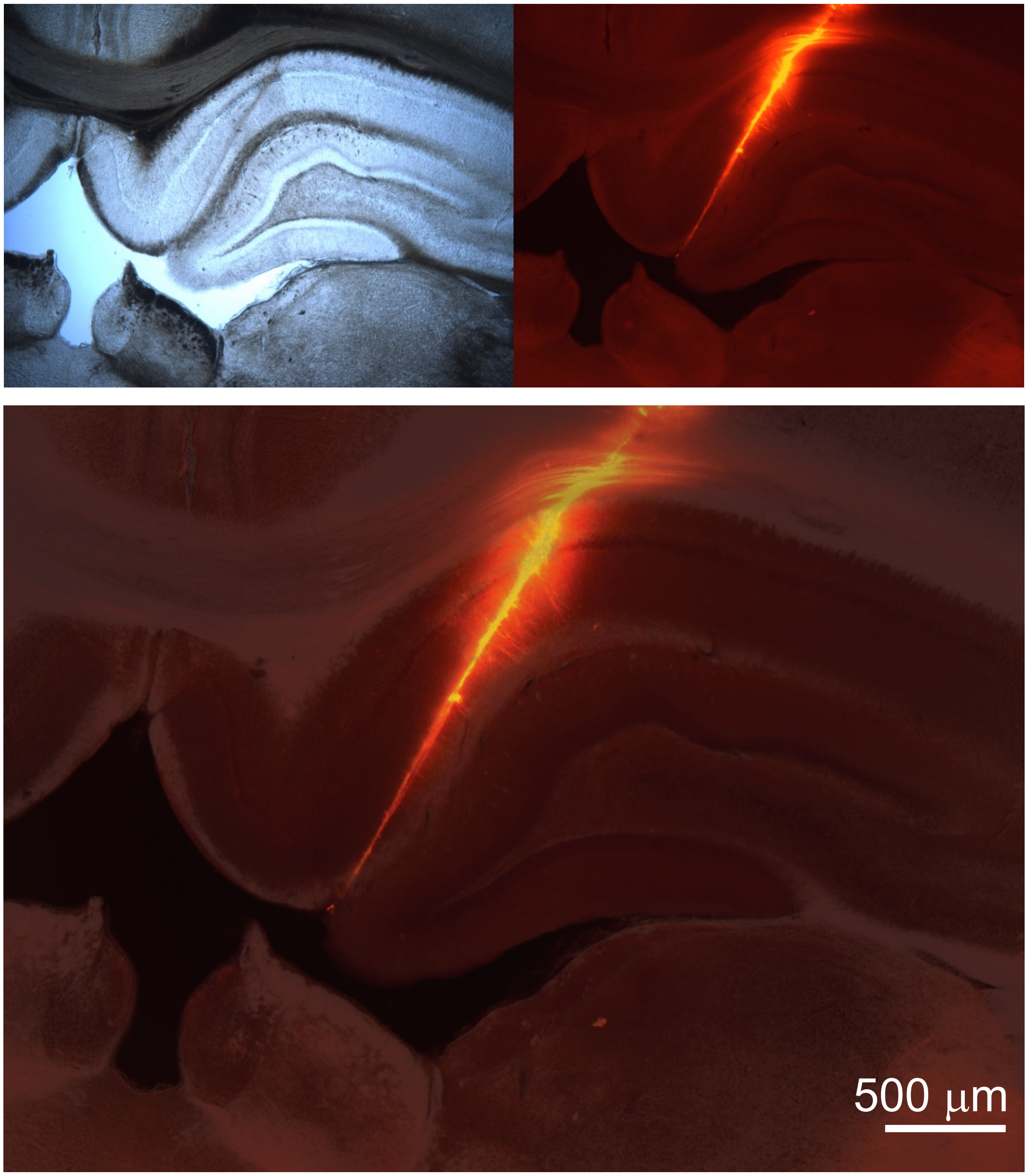

Experiment 2
Shank 1

LHb

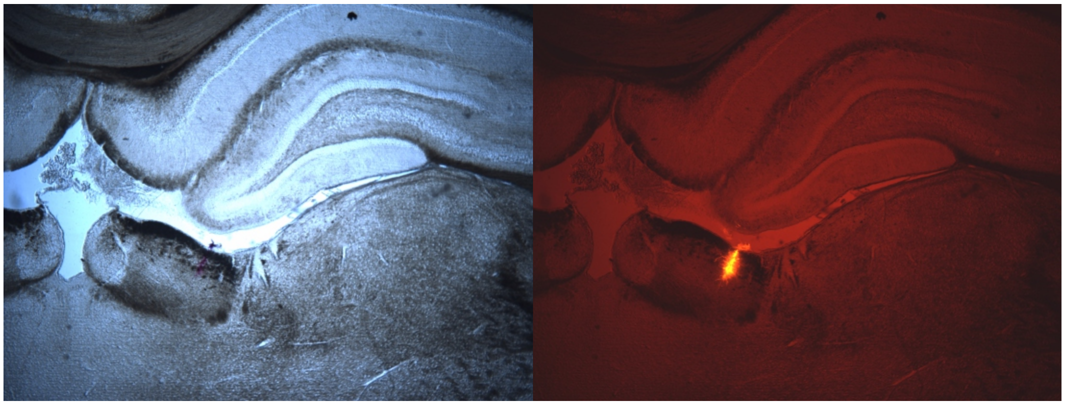

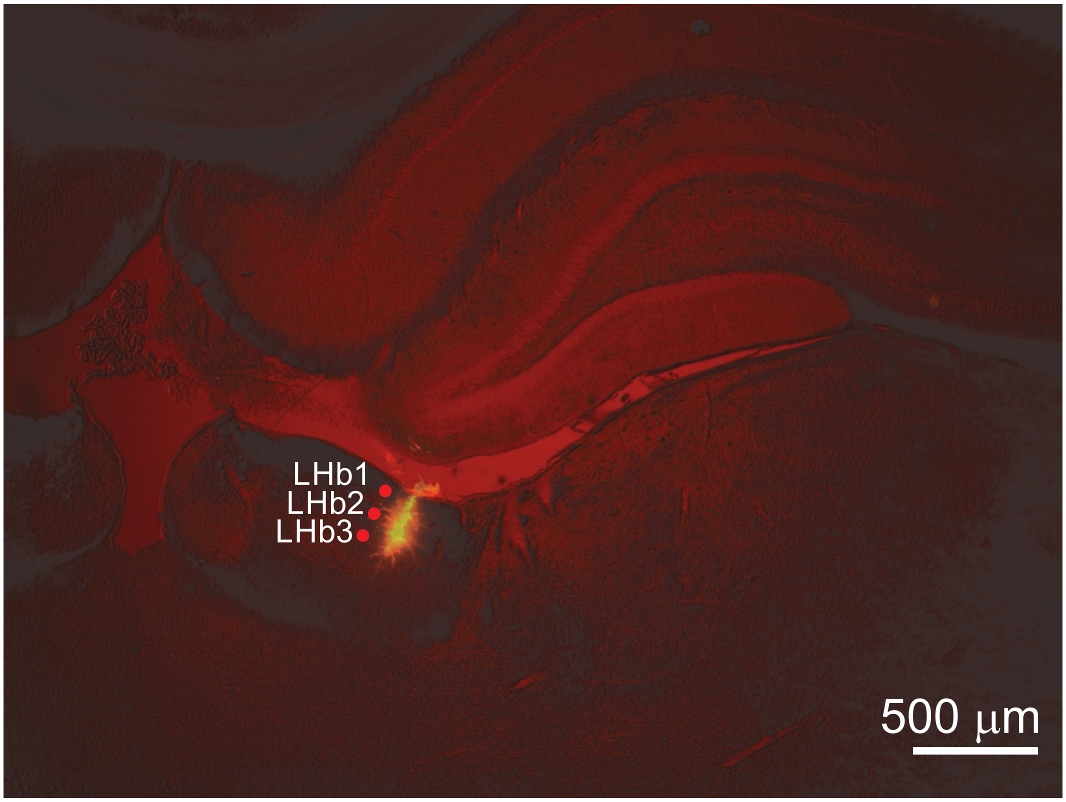

Experiment 2
Shank 1

Hippocampus

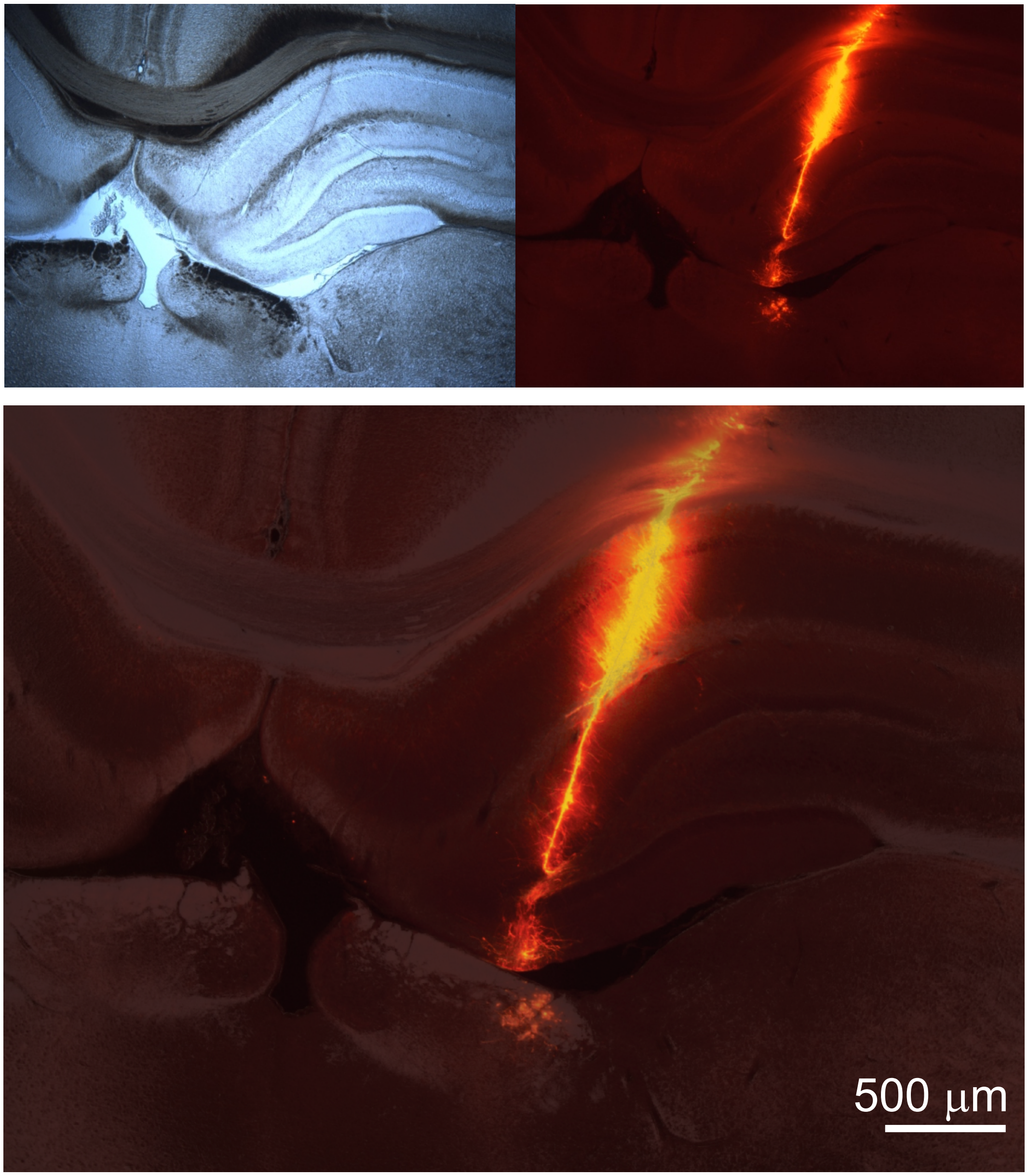

Experiment 2
Shank 2

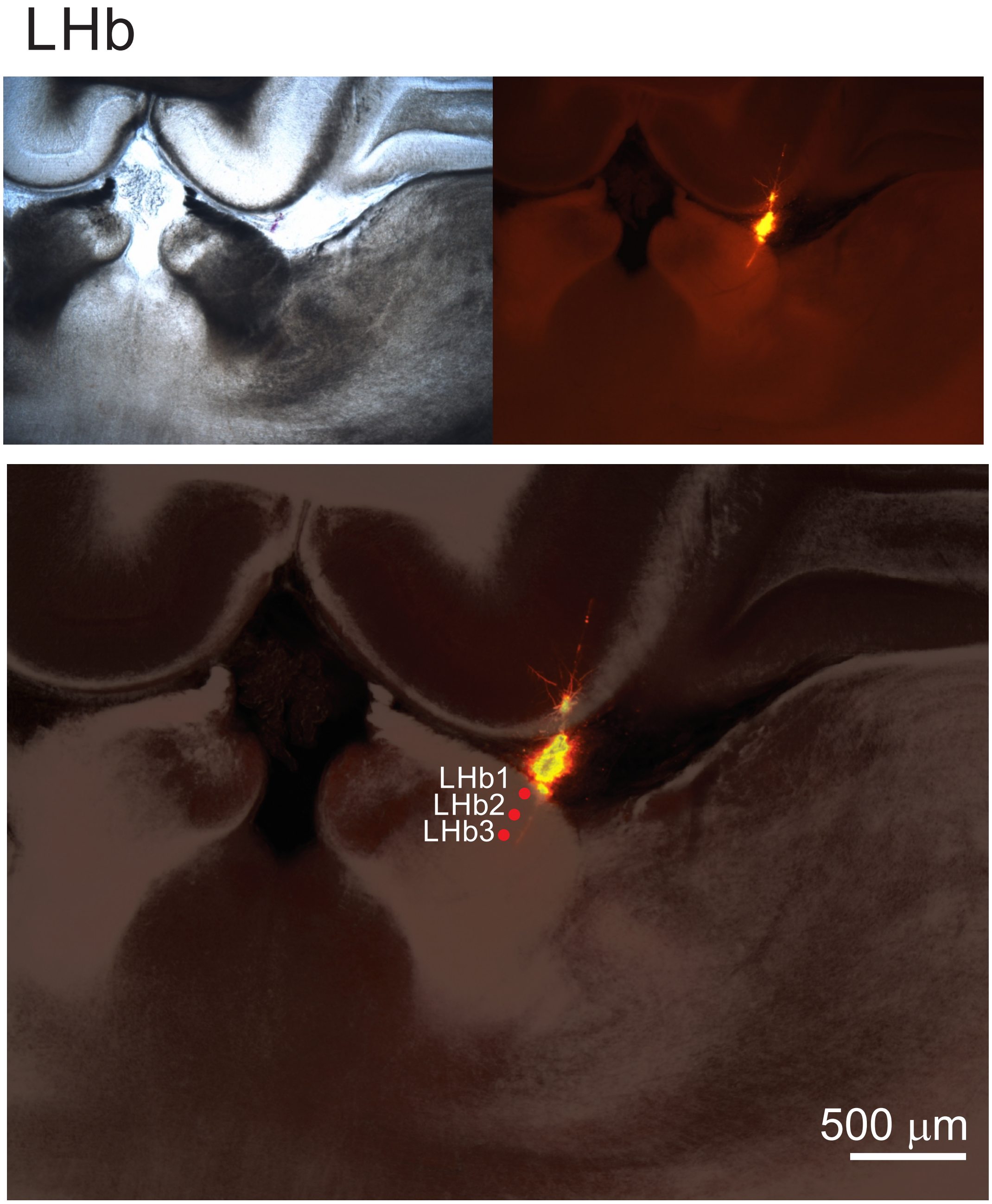

Experiment 2
Shank 2

Hippocampus

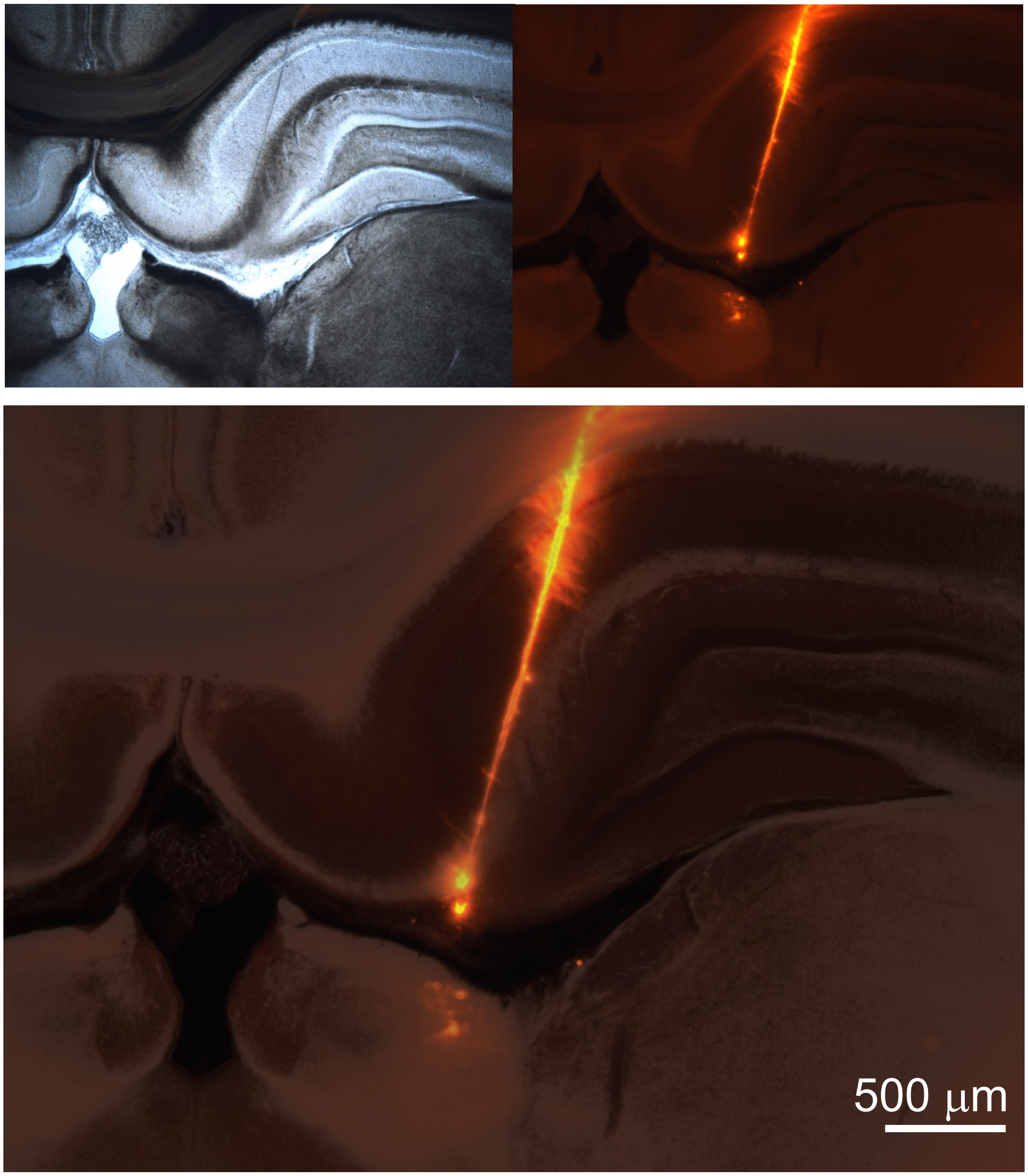

Supplement: Supplementary file 1 [file Data_Sheet_1.PDF]
