# Supplementary material for: Volume-Conducted Origin of the Field Potential at the Lateral Habenula
Source: Front Syst Neurosci. 2020 Jan 8;13:78. doi: 10.3389/fnsys.2019.00078 (PMC6961596; doi:10.3389/fnsys.2019.00078)
Supplement: Supplementary file 2 [file Data_Sheet_2.PDF]

Supplementary Figure 2. Independent component analysis of linear FPs during delta activity in experiment 1. **(A)**, wavelet spectrogram of a 30 min period of continuous recording in the st. lac-mol of the CA1. Delta and theta periods are identified by the characteristic 1 and 3-4 Hz dominant frequency, respectively (arrows). The empty and filled bars indicate the two 5 min periods used for ICA of the two electrographic states. **(B-C)**, the upper panel shows sample recordings of raw FPs along selected sites in the two shanks, and the result of the ICA analysis is shown below (temporal course) and in **C** (V-profiles). Color codes as in Fig. 3 of the main text. None of the generators show significant gradients in the LHb, hence all activity there is also volume-conducted from remote sites during delta state. Delta activity appears in a single generator (g1) with flat spatial distribution over all sites in the two recording shanks, indicating volume-conduction from outer sites. This generator accounts for >85% of total variance (**D**), and it was earlier found to arise from layers 4-6 of the cortex (Torres et al., 2019). Note that it dominates FP activity anywhere. The ICA is capable of disclosing three additional generators, two of which also have activity during theta state (compare V-profiles in Fig. 3), albeit with a different temporal pattern. These were the CA1 generator in the st. lac-mol (g_2: purple traces) and two generators that peaked in the DG (dark and light green traces). According to former studies the latter two belong to the lateral perforant path excitatory input to granule cells and granule cell somatic inhibition (Benito et al., 2014). The activity in the CA1 and DG is mostly irregular, with interspersed bouts lasting 0.5-2 seconds formed by coalescence of beta and gamma waves (see enlarged fragment in the lower panel). Colored zones in **C** denote the different regions spanned by the electrodes. **D**, relative variance contributed by each generator.

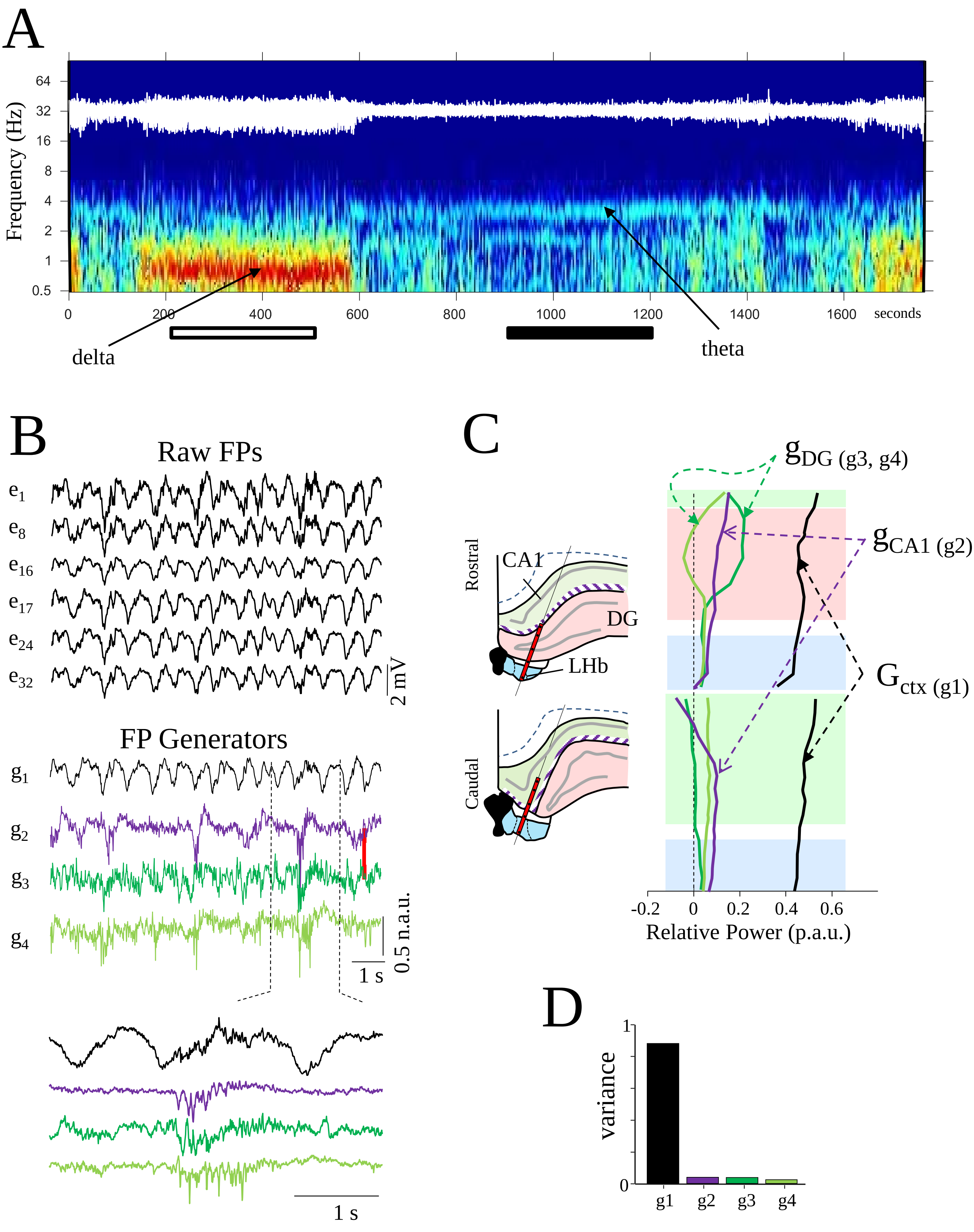

A
Frequency (Hz)
64
32
16
8
4
2
1
0.5
0
200
400
600
800
1000
1200
1400
1600
seconds
delta
theta
B
Raw FPs
e1
e8
e16
e17
e24
e32
2 mV
FP Generators
g1
g2
g3
g4
1 s
0.5 n.a.u.
1 s
C
Rostral
CA1
DG
LHb
Caudal
gDG (g3, g4)
gCA1 (g2)
Gctx (g1)
-0.2
0
0.2
0.4
0.6
Relative Power (p.a.u.)
D
variance
1
0
g1
g2
g3
g4
